# Phase Separation of FUS with Poly(ADP-ribosyl)ated PARP1 Is Controlled by Polyamines, Divalent Metal Cations, and Poly(ADP-ribose) Structure

**DOI:** 10.3390/ijms252212445

**Published:** 2024-11-20

**Authors:** Maria V. Sukhanova, Rashid O. Anarbaev, Konstantin N. Naumenko, Loic Hamon, Anastasia S. Singatulina, David Pastré, Olga I. Lavrik

**Affiliations:** 1 Institute of Chemical Biology and Fundamental Medicine (ICBFM), Siberian Branch of the Russian Academy of Sciences (SB RAS), Novosibirsk 630090, Russia; mary@niboch.nsc.ru (M.V.S.); anarbaev@niboch.nsc.ru (R.O.A.); k-naumenko@mail.ru (K.N.N.); nastsing@yandex.ru (A.S.S.); 2 INSERM U1204, Univ-Evry, University Paris Saclay, 91025 Evry, France; loic.hamon@univ-evry.fr

**Keywords:** FUS, poly(ADP-ribosyl)ated PARP1, mutant PARP1(Y986H), mutant PARP1(Y986S), microphase separation

## Abstract

Fused in sarcoma (FUS) is involved in the formation of nuclear biomolecular condensates associated with poly(ADP-ribose) [PAR] synthesis catalyzed by a DNA damage sensor such as PARP1. Here, we studied FUS microphase separation induced by poly(ADP-ribosyl)ated PARP1^WT^ [PAR-PARP1^WT^] or its catalytic variants PARP1^Y986S^ and PARP1^Y986H^, respectively, synthesizing (short PAR)-PARP1^Y986S^ or (short hyperbranched PAR)-PARP1^Y986H^ using dynamic light scattering, fluorescence microscopy, turbidity assays, and atomic force microscopy. We observed that biologically relevant cations such as Mg^2+^, Ca^2+^, or Mn^2+^ or polyamines (spermine^4+^ or spermidine^3+^) were essential for the assembly of FUS with PAR-PARP1^WT^ and FUS with PAR-PARP1^Y986S^ in vitro. We estimated the range of the FUS-to-PAR-PARP1 molar ratio and the cation concentration that are favorable for the stability of the protein’s microphase-separated state. We also found that FUS microphase separation induced by PAR-PARP1^Y986H^ (i.e., a PARP1 variant attaching short hyperbranched PAR to itself) can occur in the absence of cations. The dependence of PAR-PARP1-induced FUS microphase separation on cations and on the branching of the PAR structure points to a potential role of the latter in the regulation of the formation of FUS-related biological condensates and requires further investigation.

## 1. Introduction

Fused in sarcoma (FUS, also known as translocated in liposarcoma [TLS]), along with Ewing’s sarcoma (EWS) and TATA-binding-protein–associated factor 15 (TAF15), is a member of the highly conserved FET family of RNA-binding proteins that regulate mRNA metabolism and may be involved in DNA repair and participate in the organization of biomolecular condensates in vivo [1,2,3]. FUS belongs to the class of intrinsically disordered proteins and contains an N-terminal low-complexity domain (LCD, also called the prion-like domain), three intrinsically disordered arginine/glycine/glycine (RGG)-rich regions, a conserved RNA recognition motif (RRM), a zinc finger (ZnF) motif, and a proline-tyrosine nuclear localization signal (PY-NLS) at the C terminus [4,5]. The LCD domain and RGG-rich regions have been shown to promote the FUS phase separation that can be achieved through FUS self-assembly and/or binding to other proteins and/or nucleic acids [6,7]. For a long time, FUS has been considered a regulator of RNA metabolism [8]; nevertheless, more and more studies are revealing the direct involvement of FUS in DNA repair [2,9,10]. In this regard, DNA damage sensors such as nuclear PARPs (PARP1 and -2: members of the ADP-ribosyltransferase diphtheria toxin-like [ARTD] family) can mediate FUS participation in DNA repair events [11,12,13,14,15]. Nuclear PARP1 and PARP2 recognize damaged DNA—in particular, DNA strand breaks caused by genotoxic agents (free radicals, ionizing radiation, or alkylating agents) or by activities of repair enzymes—and catalyze the synthesis of poly(ADP-ribose) [PAR] covalently attached to proteins, mainly PARPs themselves, using NAD^+^ as a substrate [11,16]. Thus, PAR can be considered a nucleic-acid-like polymer that shares several features with single-stranded RNA or DNA, although PAR has mostly a branched-chain structure and a “star” shape [17,18]. PARP1-dependent PAR synthesis has been shown to promote the recruitment of repair enzymes and other factors to DNA lesions, thereby accelerating the repair process [19,20,21,22]. It was suggested that PAR serves as a “seed” for the promotion of FUS condensation near DNA damage [7,14,15,23,24,25].

In vitro, the recruitment of FUS to damaged DNA via binding to poly(ADP-ribosyl)ated [PARylated] PARP1 leads to the formation of large supramolecular assemblies containing damaged DNA, PARylated PARP1 (hereafter: PAR-PARP1), and FUS [15]. Moreover, FUS has been reported to form liquid-like condensates with short homogeneous and long heterogeneous protein-free PAR [7,26,27,28].

Although the FUS assembly and liquid-like phase separation (LLPS) in the presence of PAR have been actively studied in vitro [7,14,26,27,28], the features of FUS phase separation in the presence of PARylated PARPs are still not well understood due to the limited knowledge about the effect of cations. It was found that both the assembly of multimolecular compartments containing FUS, PAR-PARP1, and the damaged DNA and FUS-dependent stabilization of PARylated-PARP1–DNA condensates take place in the presence of Mg^2+^ [15,29]. Currently, physiologically relevant metal cations such as Mg^2+^ and Ca^2+^ are regarded as regulators of the LLPS of both proteins and nucleic acids [30,31]; in particular, divalent cations (Mg^2+^ or Ca^2+^) cause condensation–compaction and the microphase separation of protein-free PAR in vitro [26,28,32,33]. Moreover, we have previously observed that such cations as Mg^2+^, Ca^2+^, Mn^2+^, spermidine^3+^ (Spd^3+^), or spermine^4+^ (Spn^4+^) induce the liquid-like self-assembly of PAR-PARP1 into multimolecular associates [34,35,36].

In the present work, we attempted to elucidate how the ability of FUS to form higher-order assemblies can be implemented in the presence of PAR-PARP1. Effects of biologically relevant polyamines, of divalent cations, and of the PAR structure were investigated. By dynamic light scattering (DLS), fluorescence microscopy, atomic force microscopy (AFM), and turbidity assays, we characterized the FUS microphase separation induced by wild-type PARylated PARP1 (PAR-PARP1^WT^) or its catalytic variants PARP1^Y986S^ and PARP1^Y986H^, respectively, synthesizing (short PAR)-PARP1^Y986S^ and (short hyperbranched PAR)-PARP1^Y986H^. We then compared their properties as molecular “seeds” for the promotion of higher-order assembly of FUS. For this purpose, we conducted experiments under the conditions where FUS was primarily monomeric and its higher-order assembly was initiated by the addition of PAR-PARP1. We found that PAR-PARP1^WT^ and PAR-PARP1^Y986S^ are not able to induce FUS microphase separation. PARP1′s cofactors such as Mg^2+^, Ca^2+^, or Mn^2+^ or polyamines (Spd^3+^ or Spn^4+^) were required for the assembly of FUS with PAR-PARP1^WT^ and FUS with PAR-PARP1^Y986S^ in vitro. The range of the FUS-to-PAR-PARP1 molar ratio and cation concentration were estimated as favorable for the stability of the protein phase-separated state. We also found that the FUS microphase separation induced by PAR-PARP1^Y986H^ (a PARP1 variant synthesizing short hyperbranched PAR) can proceed in the absence of cations. The dependence of PAR-PARP1–induced FUS microphase separation on cations and the branched PAR structure points to a potential role of the latter in the regulation of the formation of FUS-related biological condensates and requires further research.

## 2. Results

### 2.1. Cations Promote the Assembly of FUS into Higher-Order Structures in the Presence of PAR-PARP1

**Figure 1:**
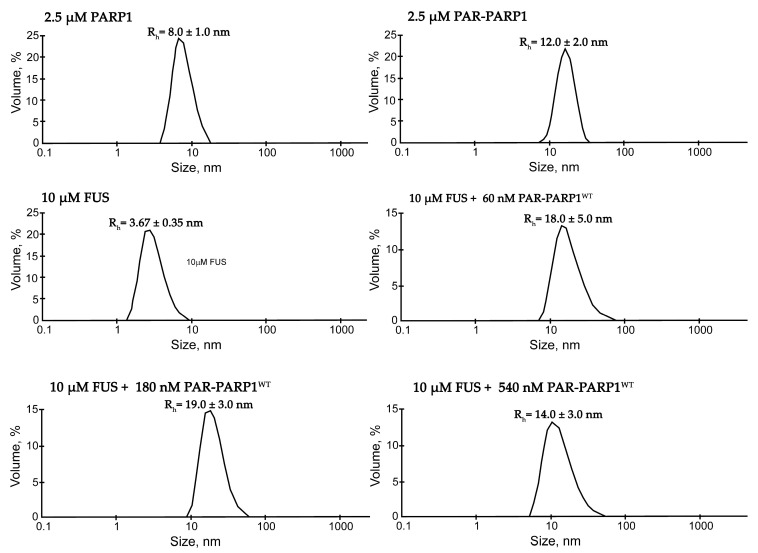
Typical volume-weighted size distributions for FUS, PARP1^WT^, PAR-PARP1^WT^, and a FUS–PAR-PARP1^WT^ mixture. The profiles were obtained by means of experimental autocorrelation functions in the Zetasizer Nano ZS software. Average hydrodynamic radii (R_h_) computed from the distributions are presented as well. R_h_ is the average value estimated from at least three DLS experiments. Size measurement was performed on FUS, PARP1^WT^, PAR-PARP1, and the mixture of FUS with PAR-PARP1^WT^ in reaction mixtures consisting of either 2.5 µM PARP1, 10 μM FUS, and 2.5 µM PAR-PARP1^WT^ or 10 μM FUS and 60–540 nM PAR-PARP1. The R_h_ values were measured directly after 3 min incubation of FUS with PAR-PARP1^WT^.

Although the mechanism that drives PAR-dependent FUS condensation is well-studied [7,14,26,27,28], little is known regarding the effects of PAR-PARP1 on the phase behavior of FUS. Earlier, by AFM, FUS was shown to interact with PAR-PARP1, thereby leading to the formation of supramolecular complexes or multimolecular compartments containing FUS, PAR-PARP1, and damaged DNA [15]. A recent study revealed that FUS stabilizes PARP1–DNA condensates, which otherwise dissociate after PARP1 PARylation [29]. In the cell, PAR mainly exists as a polymer covalently attached to proteins, mainly to PARP1 [37], and protein-free PAR can arise transiently via the hydrolysis of PAR attached to proteins [38]. Therefore, to assess the biological relevance of this phenomenon, here, we tested the propensity of FUS for liquid-like assembly under the conditions where negatively charged poly(ADP-ribose) is completely attached to PARP1, i.e., using PAR-PARP1. For this purpose, we employed DLS to monitor changes in hydrodynamic size of FUS in the presence of various concentrations of PAR-PARP1. For these experiments, we used FUS concentrations of 5–10 μM and PARP1 at ~2 μM, which are close to their physiological concentrations [7,39], and we tried to find the PAR-PARP1 concentration that promotes FUS higher-order assembly (Figure 1). We observed that PAR-PARP1 was not able to induce the assembly of FUS into higher-order structures or protein-rich microphases, even at a high FUS-to-PARP1 molar ratio, i.e., when the FUS concentration was one to two orders of magnitude higher than the PAR-PARP1 concentration (Figure 1).

Only small particles with a hydrodynamic radius of 12–19 nm were detectable under such conditions (Figure 1). The size of these particles was close to the size of PAR-PARP1 (Figure 1).

**Figure 2:**
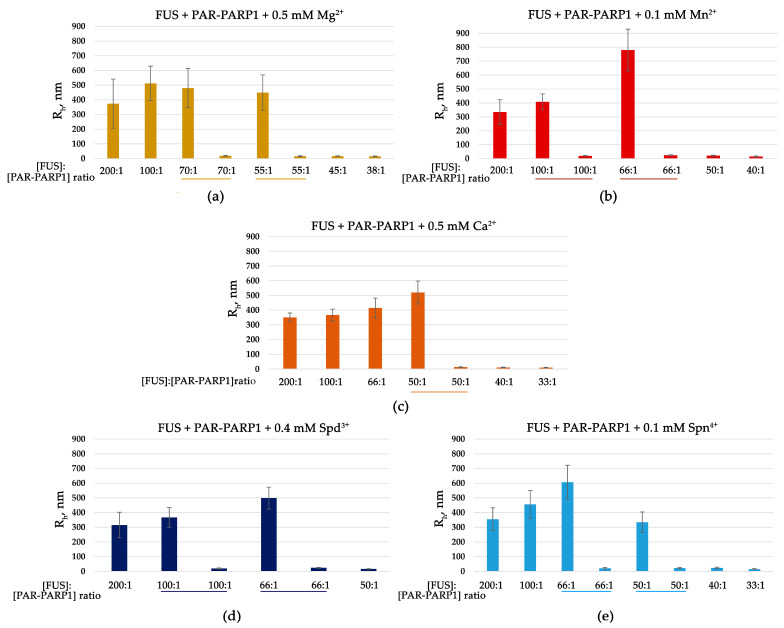
Submillimolar concentration of a cation promotes FUS microphase separation at a high FUS-to-PAR-PARP1 molar ratio. R_h_ of FUS–PAR-PARP1 mixtures is presented as a function of the FUS-to-PAR-PARP1 molar ratio. R_h_ is the average value estimated from at least three DLS experiments. Analyses of FUS higher-order structure in the presence of PAR-PARP1^WT^ were performed in reaction mixtures consisting of 10 μM FUS and 50–540 nM PAR-PARP1 as well as 0.5 mM Mg^2+^ (**a**), 0.1 mM Mn^2+^ (**b**), 0.5 mM Ca^2+^ (**c**), 0.4 mM Spd^3+^ (**d**), or 0.1 mM Spn^4+^ (**e**), as indicated in the figure. The underlining shows the FUS-to-PAR-PARP1 molar ratio at which we observed bimodal particle size distributions (Figure S1).

In our previous work, we noticed that aggregates containing FUS, PAR-PARP1, and damaged DNA arise in the presence of Mg^2+^ [15]; moreover, FUS is reported to also stabilize PAR-PARP1–DNA condensates in the presence of Mg^2+^ [29]. Therefore, we hypothesized that PAR-PARP1 could seed the FUS liquid-like assembly in the presence of Mg^2+^. Given that the cations at low concentration do not trigger the self-assembly of PAR-PARP1 [36], FUS titration with an increasing concentration of PAR-PARP1 was performed at a submillimolar concentration of Mg^2+^ or other biologically relevant cations such as Ca^2+^, Mn^2+^, Spd^3+^, or Spn^4+^ (Figure 2a–e). Indeed, the automodified PARP1 promoted the liquid-like assembly of FUS in the presence of the cations, as evidenced by the emergence of large particles with a radius of 540–834 nm (Figure 2 and Figure S1a–e).

The titration of FUS indicated that FUS higher-order assemblies arose at FUS/PAR-PARP1 molar ratios in the range of 200:1 to 50:1 (Figure 2a–e). Thus, only substoichiometric levels of PAR-PARP1 (at concentration 200- or 50-fold below that of FUS) promoted the protein assembly because, after the increase in PAR-PARP1 concentration, the assembly disappeared (Figure 2 and Figure S1a–e). A similar re-entrant phase behavior of FUS with protein-free PAR, but not with PAR-PARP1, was reported elsewhere [26,27]. Although FUS–PAR condensates are stable within a certain concentration range of PAR, they dissolve at a high PAR concentration [26,27]. Accordingly, the formation of relatively stable PAR-PARP1-FUS supramolecular assemblies was detectable at a high FUS-to-PAR-PARP1 molar ratio and after the addition of cations.

**Figure 3:**
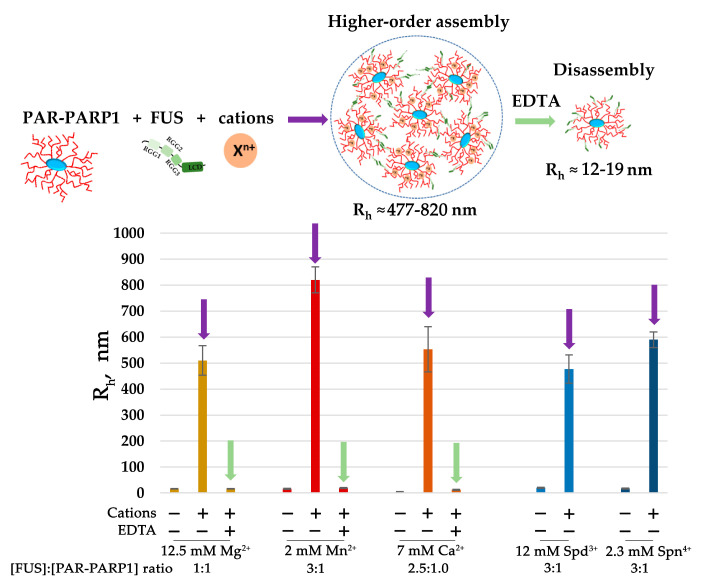
Millimolar concentration of a cation promotes FUS microphase separation at a low FUS-to-PAR-PARP1 molar ratio. FUS higher-order structure analyses in the presence of PAR-PARP1^WT^ were performed in reaction mixtures consisting of 4–5 μM FUS and 1.5–1.8 µM PAR-PARP1. PAR-PARP1 was mixed with FUS, the samples were equilibrated for 1 min, and R_h_ was measured next (Figure S2). After that, the reactions were supplemented with 2 mM Mn^2+^, 12.5 mM Mg^2+^, 7 mM Ca^2+^, 12 mM Spd^3+^, or 2.3 mM Spn^4+^, and R_h_ was measured again (Figure S2). To disrupt FUS–PAR-PARP1 assemblies stabilized by a cation, EDTA (5–30 mM) was added as indicated in the figure, and R_h_ was measured in the EDTA-treated samples (Figure S2).

Both FUS and PARP1 are abundant cellular proteins, and their intracellular concentration has been estimated as 2–8 μM for FUS and 2.0 μM for PARP1 in HeLa cells [7,39]. In our system, we also tested whether the presence of divalent cations was sufficient to induce FUS assembly when PARP1 was added at a micromolar concentration similar to that of FUS, which was within the concentrations found in the cells. By DLS measurements, we analyzed the phase behavior of the proteins within a low FUS-to-PAR-PARP1 molar range (3:1 to 1:1) and after increasing concentrations of Mg^2+^, Ca^2+^, Mn^2+^, Spn^4+^, or Spd^3+^ (Figure 3 and Figure S2). The DLS data suggested that the cations at millimolar concentration promoted the FUS assembly even when the PAR-PARP1 concentration was close to that of FUS (Figure 3). The addition of EDTA, which chelates divalent cations Mg^2+^, Ca^2+^, and Mn^2+^ [40] to the FUS–PAR-PARP1 mixture, resulted in assembly dissolution.

These data strongly support the idea that intermolecular interactions of FUS and PAR-PARP1 are stabilized by cations, even at a low FUS-to-PAR-PARP1 molar ratio that promotes the disruption of FUS assemblies (Figure 2 and Figure 3). Consequently, we supposed that the neutralization of negative charges on the PAR phosphate backbone by cations is key to FUS phase separation in the presence of PAR-PARP1. 

**Figure 4:**
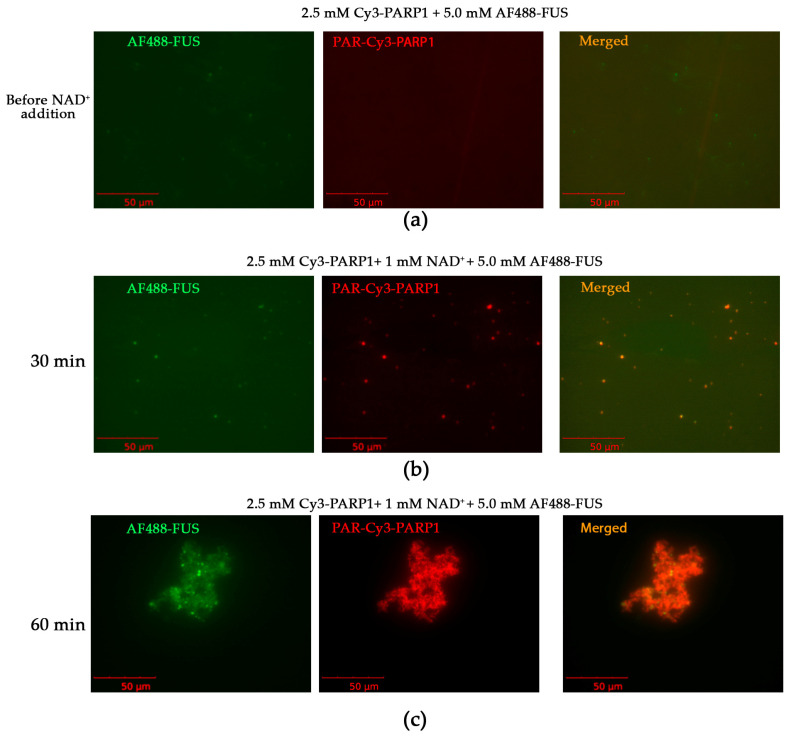
Coassembly of FUS and PAR-PARP1 in the presence of Mg^2+^. Fluorescence images of 10 µM AF488-FUS and 2.5 µM PARP1 in the presence of 15 mM Mg^2+^ (**a**) or 10 µM AF488-FUS and 2.5 µM PARP1 in the presence of 15 mM Mg^2+^ and 1 mM NAD^+^ (**b**,**c**). The fluorescence photos were captured before and after the addition of NAD^+^ to FUS–PARP1 mixtures and 30–60 min incubation at room temperature. The reaction mixtures (20 µL) contained a buffer (25 mM HEPES-NaOH pH 7.5, 200 mM NaCl, 300 nM urea, and 2 mM dithiothreitol [DTT]), 10 µM AF488-FUS, 2.5 µM Cy3-PARP1, 15 mM Mg^2+^, and 1 mM NAD^+^ as indicated.

Because these cations may promote the self-assembly of PAR-PARP1 even in the absence of FUS [36], two types of assembly may ensue: PAR-PARP1 or PAR-PARP1–FUS. Therefore, we tested by fluorescence microscopy whether FUS coassembles with PAR-PARP1. To this end, we utilized Alexa Fluor 488 (AF488)-labeled FUS and Cyanine 3 (Cy3)-labeled PARP1 and analyzed the formation of condensates at a low FUS-to-PARP1 molar ratio (4:1) to visualize both the labeled PARP1 and FUS (Figure 4). An optimal concentration of Mg^2+^ was selected to promote the phase separation (15 mM). Starting with the mixture of FUS and PARP1 at micromolar concentration (Figure 4a), we added NAD^+^, which resulted in the formation of small droplets after a 30 min incubation of the protein mixture (Figure 4b). A subsequent incubation of the mixture gave rise to larger assemblies, as evidenced by fluorescence microcopy (Figure 4c). For instance, the microscopy of the PARylated Cy3-PARP1 and AF488-FUS showed overlapping green (AF488) and red (Cy3) fluorescence signals, indicating a simultaneous presence of the two proteins in the assemblies, i.e., FUS–PAR-PARP1 coassembly in vitro (Figure 4b,c).

Overall, these experiments suggested that FUS coassembly with PAR-PARP1 can be influenced by divalent cations or polyamines. Therefore, we were able to vary the phase separation of FUS either by decreasing the PAR-PARP1 concentration or by adding cations that stabilize the condensates (Figure 2 and Figure 3). On the basis of these results, we next studied the impact of the PAR structure on the assembly formation in the PAR-PARP1–FUS system.

### 2.2. Frequency of Branching of PAR Influences the Assembly of FUS into Higher-Order Structures in the Presence of PAR-PARP1

PAR produced by PARP1 is a polymer with a branching structure and may contain up to 200 ADP-ribose units with branch points (~1%) occurring approximately every 20–50 units [17,41,42]. Taking into account that protein-free PAR’s length influences FUS condensation [26], we hypothesized that the changing of the PAR structure, namely, the formation of short, long, and/or hyperbranched polymers—when the polymer is covalently attached to PARP1—may also modulate FUS phase separation. To clarify the influence of the PAR structure on FUS’s phase behavior, we used catalytic variants of PARP1 that are a less active than the wild-type enzyme and synthesize short (PARP1^Y986S^) or short hyperbranched (PARP1^Y986H^) PAR that causes alterations in the morphology of PAR-PARP1 molecules [42,43,44]. Under our experimental conditions, the PARP1^Y986S^ variant manifested 15% residual activity relative to PARP1^WT^, whereas the PARP1^Y986H^ variant showed 50% activity (Figure S3).

**Figure 5:**
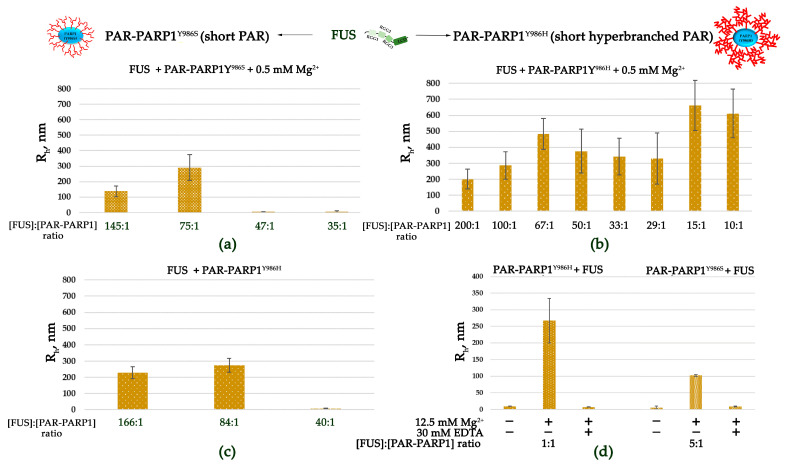
The PAR-PARP1 mutant producing hyperbranched PAR is more effective in the seeding of higher-order assembly of FUS. R_h_ is presented as a function of the FUS-to-PAR-PARP1 molar ratio. R_h_ is the average value estimated from at least three DLS experiments (Figure S5). (**a**,**b**) Analyses of FUS higher-order structure in the presence of either PAR-PARP1^Y986S^ or PAR-PARP1^Y986H^ were performed in reaction mixtures consisting of 10 μM FUS and either 60–285 nM PAR-PARP1^Y986S^ (**a**) or 50–1000 nM PAR-PARP1^Y986H^ (**b**) in the presence of 0.5 mM Mg^2+^ as indicated in the figure. (**c**) Analyses of FUS higher-order structure in the presence of PAR-PARP1^Y986H^ were performed in reaction mixtures consisting of 10 μM FUS and 60–250 nM PAR-PARP1^Y986H^. (**d**) FUS higher-order structure analyses in the presence of either PAR-PARP1^Y986S^ or PAR-PARP1^Y986H^ were performed in reaction mixtures consisting of 1.8 μM FUS and 2 µM PAR-PARP1. PAR-PARP1 was mixed with FUS, the samples were equilibrated for 1 min, and R_h_ was measured. After that, the reactions were supplemented with 12.5 mM Mg^2+^, and R_h_ was measured again. To disrupt FUS–PAR-PARP1 assemblies stabilized by a cation, EDTA (30 mM) was introduced as indicated in the figure, and R_h_ was measured in the EDTA-treated samples.

On the one hand, it was found that protein-free PAR contributes to phase separation of FUS by acting as a molecular “seed” that promotes protein assembly [7,14,26,27]. On the other hand, PAR readily disrupts FUS assemblies when present in a large molar excess over FUS [26,27]. Accordingly, a FUS–PAR system involves the phase transition of FUS from one phase to two phases and back to one phase in response to an increase in PAR concentration. This observation suggested that the less effective PAR synthesis by the PARP1 mutants may contribute to the stability of a higher-order assembly of FUS in the presence of PAR-PARP1. Therefore, we tested FUS assembly in the presence of PAR- PARP1^Y986H^ or PAR-PARP1^Y986S^. As in the case of PARP1^WT^ (Figure 1), the PARylation of either PARP1^Y986H^ or PARP1^Y986S^ did not trigger their self-assembly (Figure S4). Then, we again employed DLS to monitor changes in the hydrodynamic size of FUS at a high FUS-to-PAR-PARP1^Y986H^ (or FUS-to-PAR-PARP1^Y986S^) molar ratio and a submillimolar concentration of Mg^2+^ (Figure 5a,b and Figure S5a,b).

In the case of PARP^Y986S^ (producing short PAR), higher-order assemblies were observed at a high FUS-to-PAR-PARP1^Y986S^ molar ratio (145:1 to 75:1) (Figure 5a and Figure S5a). Thus, the FUS–PARP1^Y986S^ assembly was stable within a molar ratio that was close to the ratio observed with PARP1^WT^ (Figure 2a and Figure 5a). The results indicated that the less extensive PARP1 autoPARylation and the synthesis of shorter PAR only slightly affect the FUS-to-PARP1 molar ratio that favors the higher-order assembly of FUS with PAR-PARP1.

At the same time, FUS possesses a strong ability to form higher-order assemblies (R_h_ > 200 nm) with PAR-PARP1^Y986H^, which produces a hyperbranched polymer (Figure 5b and Figure S5b). In fact, FUS retained the capacity for microphase separation in a broad range of protein-to-PAR-PARP1^Y986H^ molar ratios (200:1 to 10:1), whereas in the cases of PAR-PARP1^WT^ and PAR-PARP1^Y986S^, large particles were detected in a narrower range of the FUS-to-PARP1 molar ratio (200:1.0 to 50:1.00) (Figure 2b and Figure 5b). Our results also revealed that the PAR-PARP1^Y986H^ variant (synthesizing hyperbranched PAR) is able to induce FUS assembly even in the absence of cations (Figure 5c and Figure S5c).

Similar to PARP1^WT^, these mutants could also promote FUS higher-order assembly at a low FUS-to-PARP1 molar ratio in the presence of Mg^2+^ when large particles with R_h_ (~267 nm) and R_h_ (~103 nm) were detectable (Figure 5d and Figure S5d). Consequently, FUS’s microphase-separated state strongly depends on the FUS-to-PAR-PARP1 molar ratio, which differed between the PARP1 variants producing PAR of different lengths and branching modes. Nevertheless, cations (Mg^2+^) were still needed to induce the assembly at a low FUS-to-PAR-PARP1^Y986H^ molar ratio (Figure 5d and Figure S5d).

**Figure 6:**
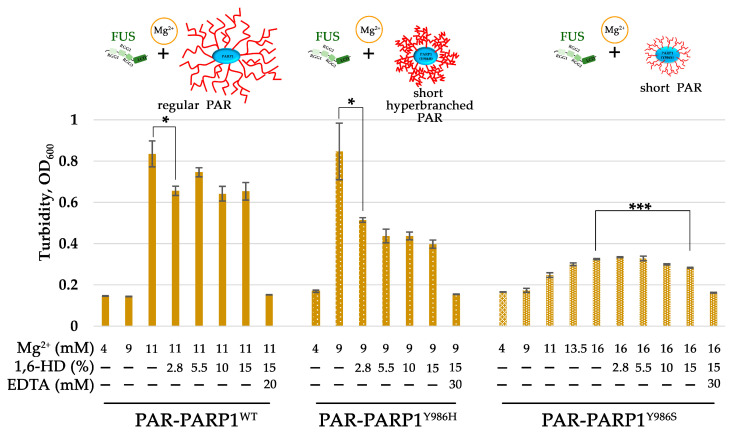
In contrast to PARP1^WT^, mutant PAR-PARP1^Y986H^ (producing hyperbranched PAR) seeds higher-order assembly of FUS at a lower concentration of Mg^2+^, and the assembly has appreciable susceptibility to 1,6-hexanediol (1,6-HD) treatment. Histograms of turbidity of a PAR-PARP1-and-FUS solution (OD at 600 nm) as determined in the presence of different concentrations of Mg^2+^, 1,6-HD, and EDTA. The mean ± SD of three independent measurements (Figures S6 and S7). *** *p* < 0.001 and * *p* < 0.05; *t* test.

To confirm that the PAR-PARP1–FUS assembly is affected by the structure of PAR, we tested whether the cation concentration (in particular Mg^2+^) needed to induce FUS microphase separation differed between PARP^WT^, PARP1^Y986S^, and PARP1^Y986H^ (Figure 6, Figures S6 and S7). For this purpose, we compared the assembly of FUS with PAR-PARP1^WT^ or its mutant in the presence of different concentrations of Mg^2+^ by measuring turbidity, which correlates with protein phase separation [45] (Figure 6, Figures S6 and S7). It was observed that, at a low FUS/PAR-PARP1 molar ratio (~1:1), 9 mM of Mg^2+^ can already promote microphase separation after the mixing of FUS and (highly branched PAR)-PARP1^Y986H^ (Figure 6). In contrast to PARP1^Y986H^, a Mg^2+^ concentration higher than 11.3 mM was required for inducing FUS microphase separation in the presence of either PAR-PARP^WT^ or PAR-PARP1^Y986S^ (Figure 6). Thus, the synthesis of highly branching PAR during PARP1 automodification required lower Mg^2+^ concentrations to promote FUS phase separation. We also tested the effect of 1,6-hexanediol: a compound widely used to disrupt weak protein–protein hydrophobic interactions underlying FUS LLPS [46].

A significant decrease in the turbidity was observed after the addition of 1,6-hexanediol to a PAR-PARP1–FUS solution, suggesting that nonionic interactions play an important part in this Mg^2+^-dependent assembly of FUS and PAR-PARP1 molecules. For a FUS–PARP1^Y986H^ mixture, an addition of 2.8% of 1,6-hexanediol diminished the turbidity twofold, suggesting that 1,6-hexanediol is efficient in disrupting the microphases (Figure 6). On the other hand, an addition of 1,6-hexanediol up to 15% to a FUS-and-PAR-PARP1^WT^ or FUS-and-PAR-PARP1^Y986S^ solution, respectively, produced only a 17% and 13% reduction in turbidity (Figure 6).

Therefore, in contrast to FUS–PAR-PARP1^WT^ or FUS–PAR-PARP1^Y986S^, the FUS–PAR-PARP1^Y896H^ assembly has higher susceptibility to dissolution by 1,6-hexanediol, indicating that this microphase-separated state depends to a lesser extent on the presence of a cation decreasing the involvement of electrostatic interactions. Accordingly, hydrophobic interactions notably contribute to FUS–PAR-PARP1^Y896H^ microphase separation. Although a noticeable reduction in turbidity by 13–50% for PAR-PARP1^WT^ and its mutants was registered at concentrations of 2.8% to 15% 1,6-hexanediol, the reagent was not able to completely disrupt FUS–PAR-PARP1 assemblies coming into being in the presence of Mg^2+^.

Only the addition of EDTA readily disrupted the assemblies, and the turbidity of the solution returned to a value similar to that of the solution with 4 mM of Mg^2+^ (Figure 6). 

These results indicated that the assembly of FUS with PAR-PARP1 is indeed a mainly cation-regulated process; however, hydrophobic interactions sensitive to 1,6-hexanediol treatment also help to stabilize the condensates.

**Figure 7:**
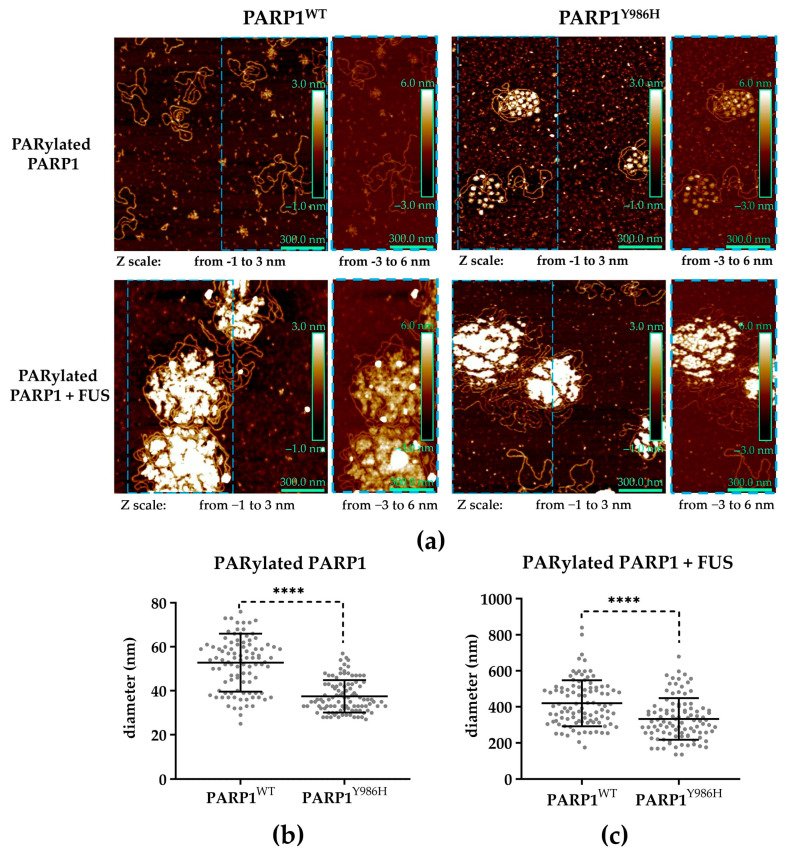
PAR structure affect the size of the higher-order assembly of FUS with PAR-PARP1. (**a**) AFM visualization of PAR-PARP1^WT^ and PAR-PARP1^Y986H^ and their higher-order assemblies with FUS. Scale bar: 300 nm; Z scale (green square): from −1 to 3 nm and from −3 to 6 nm as indicated. Blue dashed squares show the different Z scale for the same area (**b**,**c**) Measurement of the particle diameter by AFM (horizontal bars denote the mean); *n* = 100 particles from three independent samples; paired *t* test, **** *p* < 0.00001.

As reported elsewhere, mutations Y986S or Y986H in PARP1 alter both overall protein activity and the PAR chain structure [42,43,44]. Consequently, these mutations could influence the size of PAR-PARP1–FUS assemblies. To clarify this issue, we visualized a mixture of either PAR-PARP1^WT^ or PARP1^Y986H^ with FUS by AFM (Figure 7). To evaluate the size of PAR-PARP1–FUS assemblies in the presence of Mg^2+^, first, AFM imaging of PARP1^WT^ and PARP1^Y986H^ was undertaken after incubation with a gapped pBR322 (pBR) plasmid in the presence of NAD^+^ (Figure 7).

As for PARP1^WT^, the PARylated molecules had a size (diameter) up to 52 nm, whereas in the case of PARP1^Y986H^, the size was only ≤40 nm (Figure 7b). Consistent with our previous AFM data [44], modified molecules of PARP1^Y986H^ synthesizing hyperbranched PAR were found to carry highly packed polymer chains in contrast to PARP1^WT^ (Figure 7a). The activation of PARP1^WT^ and its catalytic variants as detected by AFM enabled us to measure the average size of the assemblies engendered by PARylated proteins after the addition of FUS (Figure 7c). We noticed that a decrease in the size of PAR-PARP1^Y986H^ was accompanied by a diminishing size of the assemblies that were formed by FUS in the presence of PAR-PARP1^Y986H^ (Figure 7).

Consequently, the structure of PAR, mainly its branching frequency, is an important factor for FUS higher-order assembly with PAR-PARP1 and influences the size of assemblies and the cation concentration required for the stabilization of the FUS microphase separation process.

## 3. Discussion

During the past decade, functions of RNA-binding proteins in the DNA damage response have been revised due to the discovery of biomolecular condensates generated via protein liquid–liquid phase separation [47,48]. From this point of view, the accumulation of repair proteins at DNA damage sites and the formation of DNA repair foci may be associated with condensation [7,13,14,15]. PAR and FET family proteins (FUS, EWS, and TAF15) seem to play a specific role in the formation of DNA damage-induced condensates because PAR contributes to the recruitment of FET proteins to a DNA damage site and promotes their LLPS [7,12,13,14,15]. Since the discovery of the PAR-dependent involvement of FUS in DNA repair in 2013 [12], there has been a substantial increase in the number of articles revealing the direct interactions between FUS and PARP1 and PAR [7,13,14,15,49,50]. Indeed, the emergence of FUS-rich assemblies in DNA damage regions after laser microirradiation is observed depending on PARP1 activation [7,13,14]. Moreover, FUS was shown to interact with PAR-PARP1 [49,50] and to regulate PARP1-dependent PAR synthesis in HeLa cells after genotoxic stress [50], and it possibly directly participates in the formation of repairosome compartments [14,15,29]. In vitro, FUS was found to interact with protein-free PAR, and FUS’s binding to PAR promotes the LLPS of FUS and possibly its aggregation [7,14,17,26,27,28].

In the current study, we employed a simple in vitro model of FUS phase separation that allows us to identify functional features of the interaction between FUS and PAR-PARP1 that leads to FUS microphase separation. Both free and protein-associated PAR can have similar effects on the seeding of FUS phase separation: a low PAR concentration promotes the LLPS of FUS, but when the PAR concentration exceeds a certain threshold, the condensate will begin to disintegrate [26,27]. In contrast to protein-free PAR, we failed to detect FUS assembly with PAR-PARP1 in the absence of cations; however, such bivalent cations as Mg^2+^, Ca^2+^, or Mn^2+^ or natural polyamines (Spd^3+^ or Spn^4+^) promoted the phase separation of FUS both at a high and a low FUS-to-PARP1 molar ratio (Figure 2 and Figure 3). In the presence of a submillimolar level of the cations (0.1–0.5 mM), PAR-PARP1 induced the assembly of FUS into higher-order structures in a narrow range of the FUS-to-PARP1 molar ratio (~200:1 to 50:1; Figure 2), whereas a millimolar cation concentration (2 mM Mn^2+^, 12.5 mM Mg^2+^, 7 mM Ca^2+^, 12 mM Spd^3+^, or 2.3 mM Spn^4+^) stabilized the FUS–PAR-PARP1 assembly at a low FUS-to-PARP1 ratio (~3:1 to 1:1; Figure 3). Some authors estimated that the physiological concentrations of FUS and PARP1 within HeLa cells are approximately 8.0–1.4 μM and 2.0 μM, respectively [7,39]; accordingly, under physiological conditions, PAR-PARP1 induces FUS phase separation only at its high local concentration. Nevertheless, such cations as Mg^2+^, Ca^2+^, Spd^3+^, or Spn^4+^ promoted FUS microphase separation with PAR-PARP1 even at a FUS-to-PARP1 molar ratio close to 1:1 (Figure 3 and Figure 6). Indeed, these cations are abundant in eukaryotic cells, which generally contain 1 mM polyamines, 17–20 mM of Mg^2+^, and 0.1 µM to 1 mM of Ca^2+^ [51,52,53]. Such cations as Mg^2+^, Ca^2+^, Mn^2+^, Spd^3+^, and Spn^4+^ play an important part in the regulation of many RNA- and DNA-dependent processes and can serve as cofactors of PARP1 [32,54,55,56,57,58]. Moreover, some of these cations (Mg^2+^, Ca^2+^, and Spn^4+^) are reported to regulate the formation of membraneless organelles, to modulate the phase behavior of both intrinsically disordered proteins and RNA, DNA, and PAR, which are key components of cellular protein condensates and aggregates [30,31,59]. Moreover, we have shown recently that cations are able to promote the liquid-like self-assembly of PARylated PARP1 in the absence of FUS [36].

Here, we demonstrated that the near-physiological concentration of the cations tested in our experiments has a strong impact on the phase behavior of FUS in the presence of PAR-PARP1 in vitro. The present work also addresses effects of the PAR structure on FUS phase separation. As mentioned above, protein-free PAR induces FUS LLPS at a low concentration but dissolution at a high concentration [26,27]. Furthermore, it was expected here that PAR-PARP1—similarly to protein-free PAR—would reversibly modulate the microphase separation of FUS, but modified PARP1 was unable to trigger FUS assembly without cations (Figure 1). Given that the noncovalent FUS–PAR interaction and FUS assembly are tightly linked within the phase separation process [7,14,15,26,27,28], PAR structure, namely, length and branching frequency, may be regarded as a key factor for the regulation of FUS phase separation [26,33]. To explore possible relations between FUS assembly and the PAR structure produced by PARP1, we also investigated the properties of PARP1 catalytic variants PARP1^Y986S^ and PARP1^Y986H^ in the “seeding” of higher-order assembly of FUS (Figure 5, Figure 6 and Figure 7). There are currently no data showing that these mutations are inherited and naturally occurring [60], but these mutations strongly affect PAR structure and the morphology of PARylated PARP1 molecules, allowing functional effects of PAR structure to be observed in the reconstituted systems [42,44,61]. We found that the point mutations in PARP1—that cause both a reduction in the magnitude of PARP1 autoPARylation and changes in the structure of PAR—strongly influence the FUS assembly; this effect was especially evident in the case of PARP1^Y986H^, which produces short hyperbranched PAR (Figure 5 and Figure 6). In the experiments with FUS and the PARP1 mutants, we noted that it was not the PAR length but the level of PAR branching that is the main factor that influences the assembly of FUS into higher-order structures in the presence of PAR-PARP1 (Figure 5, Figure 6 and Figure 7). This finding implies that the promotion of FUS microphase separation substantially depends on the structure of PAR because hyperbranched PAR is more effective than long or short PAR having regular or slightly enhanced branching, as in the case of PARP1^WT^ and its PARP1^Y986S^ mutant (Figure 2 and Figure 5). In the presence of Mg^2+^, (hyperbranched short PAR)-PARP1^Y986H^ and (short PAR)-PARP1^Y986S^ (or PAR-PARP1^WT^) can induce FUS microphase separation at a FUS-to-PARP1 molar ratio differing by a factor of five (Figure 2a and Figure 5a,b). Moreover, hyperbranched PAR-PARP1^Y986H^ seeded FUS microphase separation in the absence of cations (Figure 5c). Thus, we believe that the PAR-induced assembly and disassembly of FUS higher-order structures are affected by PAR’s structural characteristics such as branching and length.

The observation that PAR binders such as cell cycle regulators, repair proteins, and histones, show a distinct preference for interacting with PAR of different chain lengths and branching led to the hypothesis of the existence of a ‘PAR code’ in which different PARylation sites on the proteins and the length and structure of the polymer linked to a particular protein target are involved in the regulation of cellular processes [21,23,62,63,64]. The interaction of various proteins with PAR have been well characterized in numerous in vitro studies [21,44,61,63], and few studies have shown an obvious function of PAR chain length and branching frequency in the regulation of the cellular stress response [42,60,65]. Although the association of PARP1 PARylation with the phase separation of FUS and other FET proteins has been demonstrated [7,14], little is known about the effect of PAR structure on the phase separation behavior of FUS [26]. Taking into account that (hyperbranched PAR)-PARP1^Y986H^ has an obvious preference for the seeding of FUS phase separation (Figure 5b), the PAR structure may perform a pivotal function in the modulation of formation efficiency of FUS-dependent condensates associated with PARP1 activation at DNA damage sites.

**Figure 8:**
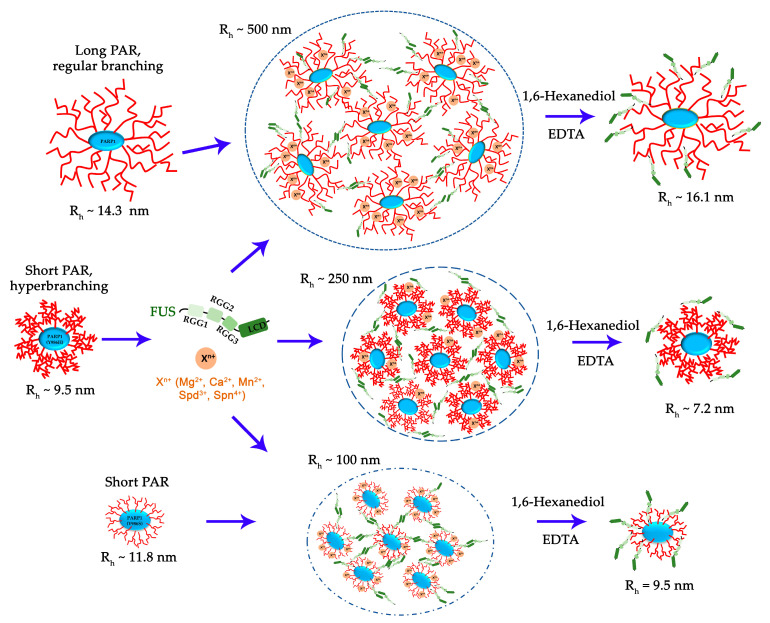
The proposed model of cation-dependent assembly of FUS with PAR-PARP1. Cations stabilize FUS association with PAR-PARP1; such assembly is sensitive to chelating agents such as EDTA and to 1,6-hexanediol, which disrupts hydrophobic interactions.

This relation also opens up new opportunities for a better understanding of the mechanisms by which PAR-PARP1 and PAR structures can influence FUS phase separation and should elucidate the potential function of cations in the regulation of FUS-dependent condensate formation (Figure 8). Moreover, we recently showed that PAR-PARP1 undergoes self-assembly into supramolecular complexes in the presence of cations; in addition, we observed that the self-assembly of PARP1 regulates the level of its automodifications and stimulates activities of poly(ADP-ribose)glycohydrolase to hydrolyze PAR and DNA polymerase β, a key DNA polymerase of base excision repair and DNA single-strand repair pathways [36]. At the same time, there are data about the PAR-dependent organization of biomolecular condensates with the participation of FUS both in vitro and ex vivo [7,14,15,26,27]. Additional research will clarify whether FUS condensation and FUS’s interaction with PAR-PARP1 have functional consequences, such as the regulation of PARP1-dependent DNA repair pathways or the activity of PAR-degrading enzymes.

In this way, the results of this study may advance the current knowledge of the mechanisms of biomolecular condensate formation involving a key DNA damage sensor, PARP1 [66], and the RNA binding protein FUS, whose cellular functions are closely associated with neurodegenerative diseases [67]. Further investigation of how PARylated PARP1 orchestrates FUS phase separation will deepen our understanding of neurological diseases and cancers caused by mutations in these proteins and may provide the basis for the development of innovative technologies in cancer therapy.

## 4. Materials and Methods

### 4.1. Plasmid Construction, Proteins, and Reagents

Plasmid pET32a-hPARP1-His encoding cDNA of wild-type recombinant human PARP1 with a His tag is a kind gift from Dr. M. Satoh (Université Laval, Québec, QC, Canada). pET32a-hPARP1-His was used to generate mutation Y986S or Y986H within the *PARP1* coding sequence [44].

The recombinant wild-type PARP1 (PARP1^WT^) and mutants PARP1^Y986S^ and PARP1^Y986H^ were overexpressed in *Escherichia coli* (*E. coli*) Rosetta (DE3)pLysS (Novagen, Darmstadt, Germany) and purified by means of Ni-NTA agarose (GE Healthcare, Milwaukee, WI, USA) affinity chromatography, HiTrap Heparin High Performance (GE Healthcare, Milwaukee, WI, USA) affinity chromatography, and deoxyribonucleic-acid−cellulose (single-stranded calf thymus DNA) (Sigma-Aldrich, St. Louis, MO, USA) affinity chromatography as described before [44]. Recombinant FUS was expressed in *E. coli* strain BL21(DE3) and purified as described previously [15].

Sulfo-Cy3 NHS ester and AF488 NHS ester were purchased from Lumiprobe RUS Ltd. (Moscow, Russia). Radioactive [α-^32^P]ATP was prepared in the Laboratory of Biotechnology at the ICBFM (SB RAS, Novosibirsk, Russia). Oligodeoxynucleotides were synthesized by the Laboratory of Biomedical Chemistry (ICBFM SB RAS, Novosibirsk, Russia). NAD^+^ and β-nicotinamide mononucleotide were purchased from Sigma-Aldrich (St. Louis, MO, USA), and the pBR plasmid were purchased from New England BioLabs (Ipswich, MA, USA). Reagents for buffers and electrophoresis components were bought from Sigma-Aldrich (St. Louis, MO, USA), PanReacAppliChem (Darmstadt, Germany) (acrylamide/bis-acrylamide and urea), Molecular Group (dithiothreitol [DTT]), and Merk (NaCl), whereas protein molecular weight markers were purchased from ThermoScientific (Vilnius, Lithuania). Olaparib (AZD2281, Ku-0059436) was purchased from Apexbio Technology (Houston, TX, USA).

### 4.2. Preparation of the DNA Duplex and Damaged Plasmid

A DNA duplex (30-mer with a one-nucleotide gap) (DNA-gap) was obtained via the hybridization of an oligonucleotide (3′-cccaaccaaacgc g taagtgtcaagaggcg-5′) with complementary oligonucleotides (5′-OH-gggttggtttgcg-3′ and 5′-phosphate-attcacagttctccgc-3′) in a 1.0:1.5 ratio. The oligonucleotide mixture was incubated for 3 min at 95 °C and then slowly cooled to room temperature.

The pBR plasmid-containing single-strand DNA breaks was prepared using heat and acid treatment to create abasic sites followed by apurinic/apyrimidinic-site cleavage by means of the apurinic/apyrimidinic endonuclease 1 activity as described previously [15].

### 4.3. Preparation of PAR-PARP1

PAR-PARP1 was prepared in a reaction mixture (20 µL) consisting of 2.5 µM PARP1^WT^ (or its mutant PARP1^Y986S^ or PARP1^Y986H^), 2.0 µM DNA duplex, 200 mM NaCl, 300 mM urea, 25 mM HEPES-NaOH pH 7.5, and 1 mM DTT. Samples were equilibrated for 1 min, and then the PARP1 activation was initiated by the addition of NAD^+^ to a final concentration of 1 mM. The reaction mixtures were incubated at 30 °C for 40 min. The reactions were stopped by the addition of olaparib to a final concentration of 200 µM.

### 4.4. Hydrodynamic Size Measurement by DLS

DLS measurements were carried out in a low-volume quartz batch cuvette (ZEN 2112) using a Zetasizer Nano ZS instrument (Malvern Instruments Ltd., Malvern, UK) and Zetasizer Software 7.11 at 25 °C. All stock solutions of DNA and proteins were filtered through a polyethersulfone membrane (0.2 μm pore size). The measurements and data processing were performed as described elsewhere [34,36].

FUS, PARP1^WT^, PARP1^Y986H^, and PARP1^Y986S^ hydrodynamic size assays were performed in reaction mixtures consisting of a DLS buffer (25 mM HEPES-NaOH pH 7.5, 200 mM NaCl, 300 mM urea, and 1 mM DTT), 10 μM FUS, 2.5 µM PARP1^WT^, 2.5 µM PARP1^Y986H^ or 2.5 µM PARP1^Y986S^ as specified in figure legends. The samples were equilibrated at 30° for 1 min, and then the R_h_ measurement was performed.

For analysis of the PAR-PARP1^WT^, PAR-PARP1^Y986H^, or PAR-PARP1^Y986S^ hydrodynamic size, 2.5 µM PARP1^WT^, PARP1^Y986H^, or PARP1^Y986S^ was incubated with 2.5 µM DNA-gap in the DLS buffer. Samples were equilibrated for 1 min, after which PARP1 activation was initiated by the addition of NAD^+^ to a final concentration of 1 mM. The reaction mixtures were incubated at 30 °C for 40 min, and next R_h_ measurement was performed.

For the analysis of hydrodynamic size at a high FUS-to-PARP1 molar ratio, 10 µM FUS was mixed with 50–650 nM PAR-PARP1^WT^, PARP1^Y986S^, or PARP1^Y986H^ in a DLS buffer (25 mM HEPES-NaOH pH 7.5, 200 mM NaCl, 300 mM urea, and 1 mM DTT) in the absence or presence of 0.1 mM Mn^2+^, 0.1 mM Spn^4+^, 0.4 mM Spd^3+^, 0.5 mM Ca^2+^, or 0.5 mM Mg^2+^ as indicated in figure legends. The samples were equilibrated at 30 °C for 1 min, after which R_h_ was measured.

For the analysis of hydrodynamic size at a low FUS-to-PARP1 molar ratio, 1.0–1.5 µM PAR-PARP1 was mixed with 5–4 µM FUS in a DLS buffer, the samples were equilibrated for 1 min, and R_h_ was measured. After that, the reactions were supplemented with Mn^2+^ up to 2 mM, Mg^2+^ up to 12.5 mM, Ca^2+^ up to 7 mM, Spd^3+^ up to 12 mM, or Spn^4+^ up to 2.3 mM. The samples were equilibrated at 30 °C for 1 min, and R_h_ was measured again.

### 4.5. Turbidity Measurements

For assessment of the turbidity of FUS solutions in the presence of PAR-PARP1, 1.8 µM FUS was incubated with 2 µM PAR-PARP1^WT^, PAR-PARP1^Y986S^, or PAR-PARP1^Y986H^ in a buffer consisting of 100 mM NaCl, 20 mM HEPES-NaOH pH 7.5, and 300 mM urea at 30 °C for 5 min. After that, the reactions were supplemented with 4–16 mM Mg^2+^, as indicated in the figure legend, and an absorption spectrum of the solutions was recorded. Then, either 1,6-hexanediol (2.8–15.0%) or EDTA (30 mM) was introduced as indicated in the figure legend, and the absorption spectrum of the solutions was recorded again.

### 4.6. Fluorescent Labeling of FUS and PARP1 and Fluorescence Microscopy

For the labeling of FUS, purified FUS (19 nmol, 4.6 mg/mL) was incubated with AF488 (45 nmol) for 1 h at 25 °C and then at 4 °C overnight in a buffer consisting of 6 mM HEPES-NaOH pH 7.5, 50 mM NaCl, 2.0 M urea, and 100 mM NaHCO_3_. The unreacted AF488 dye was removed via dialysis against a buffer composed of 20 mM HEPES-NaOH pH 7.5, 200 mM NaCl, 6 M urea, and 1 mM DTT followed by a concentration of the AF488-labeled FUS (AF-FUS) in ultrafiltration spin columns. For the labeling of PARP1, purified PARP1 (1.2 nmol, 3.0 mg/mL) was incubated with Cy3 (13 nmol) for 1 h at 25 °C and then at 4 °C overnight in a buffer consisting of 50 mM HEPES-NaOH pH 7.5, 400 mM NaCl, and 2 mM tris(2-carboxyethyl)phosphine (TCEP). The unreacted Cy3 dye was removed via dialysis against a buffer composed of 50 mM HEPES-NaOH pH 7.5, 200 mM NaCl, and 1 mM TCEP followed by a concentration of the Cy3-labeled PARP1 (Cy3-PARP1) in ultrafiltration spin columns.

Concentrations of the conjugates AF488-FUS and Cy3-PARP1 and the degree of labeling (DOL) were determined using the following extinction coefficients: ε_280_ = 70,390 M^−1^cm^−1^ for FUS, ε_280_ = 120,000 M^−1^cm^−1^ for PARP1, ε_555_ = 150,000 M^−1^cm^−1^, and a correction factor (CF_280_) of 0.09 for Cy3, ε_495_ = 71,800 M^−1^cm^−1^, and CF_280_ = 0.1 for AF488. The DOL was calculated via the formula

DOL = A_max_ × ε_prot_/((A_280_ − A_max_ × CF_280_) × ε_280_), (1)

where ε_prot_ and ε_max_ are the molar extinction coefficients of the protein and dye, respectively; A_max_ is at the absorption maximum of the dye; and A_280_ is absorbance of the protein at 280 nm.

The DOL was estimated at 12% for FUS and 90% for PARP1. The extinction coefficients (ε_prot_) of FUS and PARP1 were based on Expasy Protparam data, and the extinction coefficients (ε_max_) for AF488 and Cy3 were taken from Lumiprobe protocols.

### 4.7. Preparation of Samples for AFM Experiments and Image Analysis

Complexes of PAR-PARP1 and FUS for AFM were generated in reaction mixtures (20 μL) composed of 12.5 mM of HEPES-NaOH pH 8.0, 12.5 mM NaCl, 1 mM DTT, 5 mM MgCl_2_, 100 mM urea, either 30 nM PARP1^WT^ or PARP1^Y986H^, and 12.5 nM damaged pBR. The samples were equilibrated for 1 min, and then the PARP1 activation was initiated by the addition of NAD^+^ to a final concentration of 0.3 mM, with incubation at 37 °C for 5 min, after which FUS was introduced at a final concentration of 400 nM, followed by incubation at 37 °C for 1 min. Next, the samples were diluted 10-fold with a buffer (12.5 mM HEPES-NaOH pH 8.0, 12.5 mM NaCl, and 1 mM DTT). To adsorb the molecules on mica, putrescine was added to the solution to a final concentration of 1 mM, after which a 10 μL droplet was deposited on the surface of freshly cleaved mica and processed and imaged as described before [15,18]. AFM images were captured in air using a Nanoscope V Multimode 8 (Bruker, Santa Barbara, CA, USA) in PeakForce Tapping mode using Scanasyst-Air probes (Bruker). Continuous force–distance curves were thus recorded with an amplitude of 100–300 nm at low frequency (1–2 kHz). PeakForce Tapping mode decreases lateral and shear forces. Images were captured at 2048 × 2048 pixels at a line rate of 1.5 Hz.

The minimum diameter (nm) of the circle in which PARylated proteins or complexes could be enclosed was measured in AFM images with the help of the “section” tool in the Nanoscope Analysis software (version 1.70).

## 5. Conclusions

The recent discovery of the PAR-dependent liquid–liquid phase separation of FUS has led to numerous studies of FUS-PARP1 and FUS-PAR interactions in response to DNA damage using in vitro and ex vivo systems [7,12,13,14,15,26,27,28,29,50]. Future research using animal models is needed to further our understanding of the PARP-dependent mechanism of FUS condensate formation in the nucleus, as well as FUS functions downstream of nuclear PARPs, such as the involvement of FUS in the organization of DNA repair compartments, the assembly of stress granules or the accumulation of cytoplasmic FUS inclusions, which are a hallmark of frontotemporal lobar degeneration and amyotrophic lateral sclerosis.

## Data Availability

The original contributions presented in this study are included in the article and Supplementary Materials; further inquiries can be directed to the corresponding author.

## Supplementary Materials

The following supporting information can be downloaded at: https://www.mdpi.com/article/10.3390/ijms252212445/s1.
