# Supplementary material for: Phase Separation of FUS with Poly(ADP-ribosyl)ated PARP1 Is Controlled by Polyamines, Divalent Metal Cations, and Poly(ADP-ribose) Structure
Source: Int J Mol Sci. 2024 Nov 20;25(22):12445. doi: 10.3390/ijms252212445 (PMC11594298; doi:10.3390/ijms252212445)
Supplement: Supplementary file 1 [file ijms-25-12445-s001.zip › ijms-3256547-supplementary.pdf]

Supplementary Materials

Supplementary Figures

FUS + PAR-PARP1^{WT} + 0.5 mM Mg^{2+}

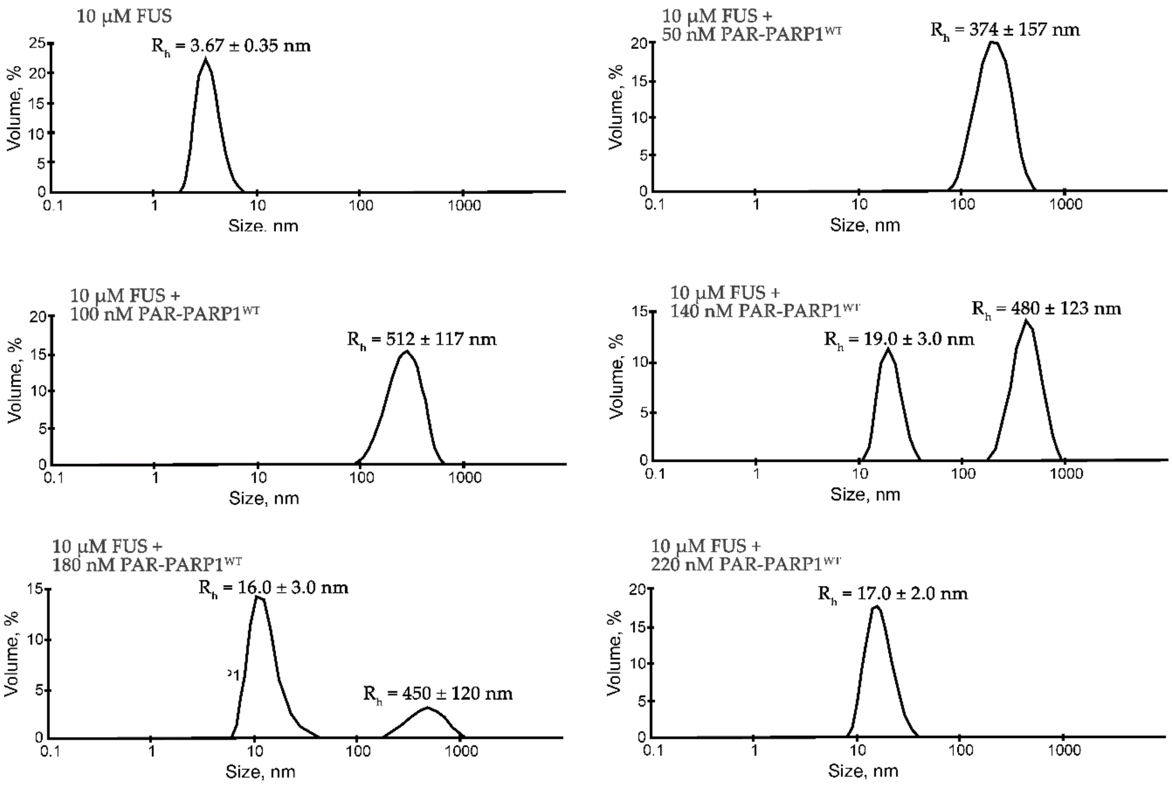

Figure S1a. Typical volume-weighted size distributions for the mixture of FUS with PAR-PARP1^{WT} in the presence of Mg^{2+}. The profiles were obtained by means of experimental autocorrelation functions in the Zetasizer Nano ZS software. The average hydrodynamic radii (R_h) computed from the distributions are presented as well. Rh is the average Rh value estimated from at least three DLS experiments.
FUS higher-order structure assays in the presence of PAR-PARP1^{WT} were performed in reaction mixtures consisting of 10 µM FUS, 50-220 nM PARylated PARP1 and 0.5 mM Mg^{2+} in DLS buffer consisting of 25 mM HEPES-NaOH pH 7.5, 200 mM NaCl, 300 mM urea, and 1 mM dithiothreitol (DTT). The R_h values were measured directly after 1-min incubation of FUS with PAR-PARP1^{WT}.

FUS + PAR-PARP1WT + 0.1 mM Mn^{2+}

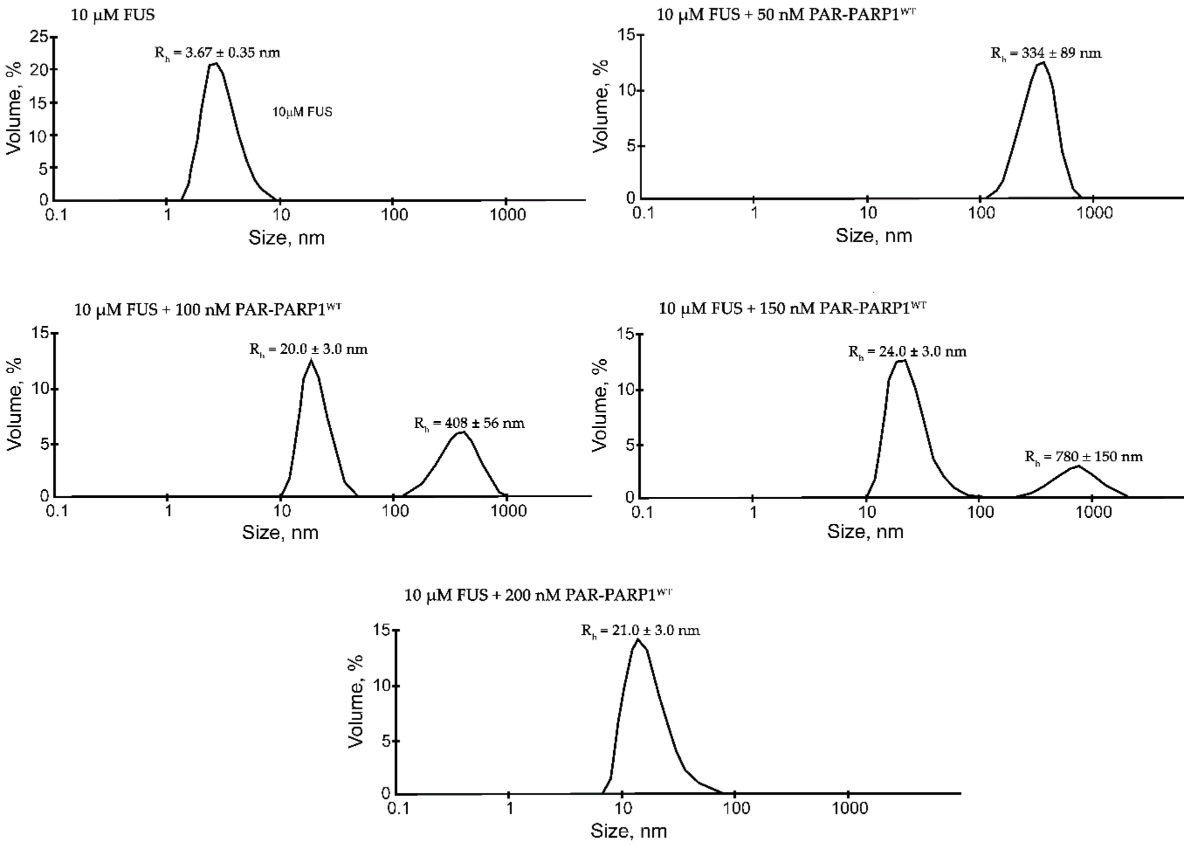

Figure S1b. Typical volume-weighted size distributions for the mixture of FUS with PAR-PARP1^{WT} in the presence of Mn^{2+}. The profiles were obtained by means of experimental autocorrelation functions in the Zetasizer Nano ZS software. The average hydrodynamic radii (R_h) computed from the distributions are presented as well. R_h is the average Rh value estimated from at least three DLS experiments.

FUS higher-order structure assays in the presence of PAR-PARP1^{WT} were performed in reaction mixtures consisting of 10 µM FUS, 50-220 nM PARylated PARP1 and 0.1 mM Mn^{2+} in DLS buffer consisting of 25 mM HEPES-NaOH pH 7.5, 200 mM NaCl, 300 mM urea, and 1 mM DTT. The Rh values were measured directly after 1-min incubation of FUS with PAR-PARP1^{WT}.

FUS + PAR-PARP1^{WT} + 0.5 mM Ca^{2+}

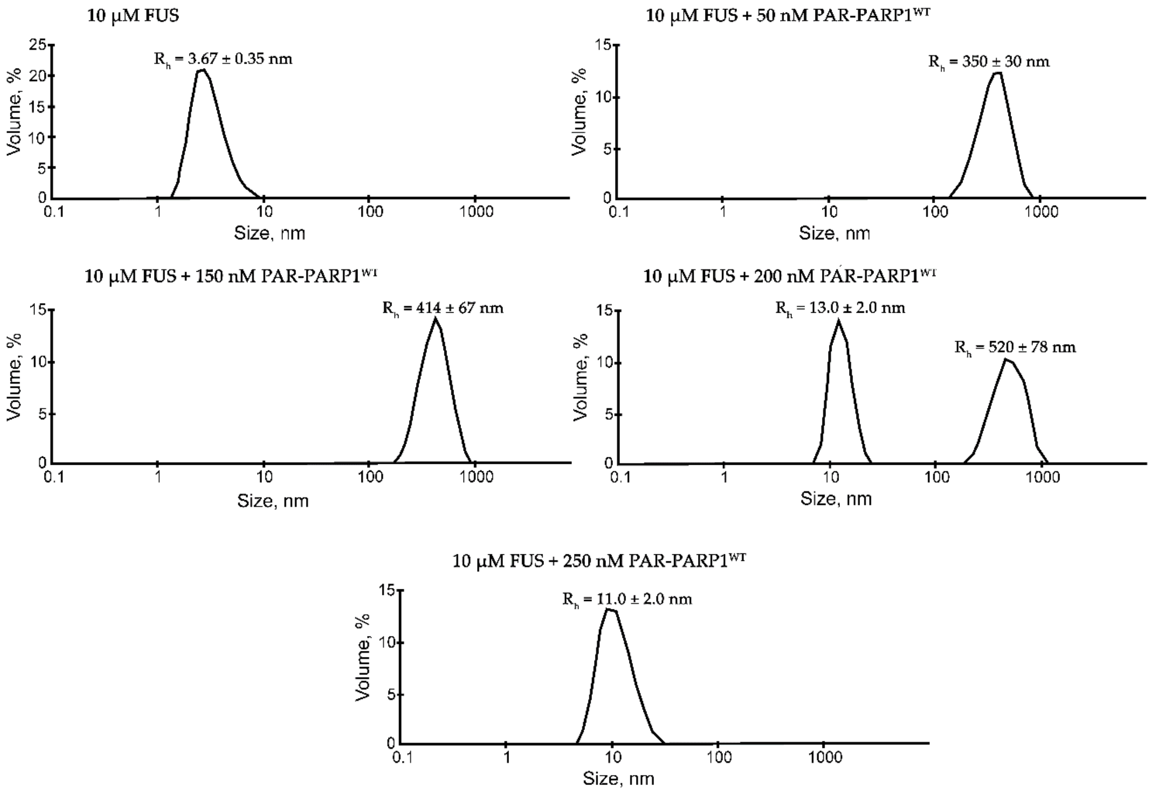

Figure S1c. Typical volume-weighted size distributions for the mixture of FUS with PAR-PARP1^{WT} in the presence of Ca^{2+}. The profiles were obtained by means of experimental autocorrelation functions in the Zetasizer Nano ZS software. The average hydrodynamic radii (R_h) computed from the distributions are presented as well. Rh is the average Rh value estimated from at least three DLS experiments. FUS higher-order structure assays in the presence of PAR-PARP1^{WT} were performed in reaction mixtures consisting of 10 µM FUS, 50-250 nM PARylated PARP1 and 0.5 mM Ca^{2+} in DLS buffer consisting of 25 mM HEPES-NaOH pH 7.5, 200 mM NaCl, 300 mM urea, and 1 mM DTT. The R_h values were measured directly after 1-min incubation of FUS with PAR-PARP1^{WT}.

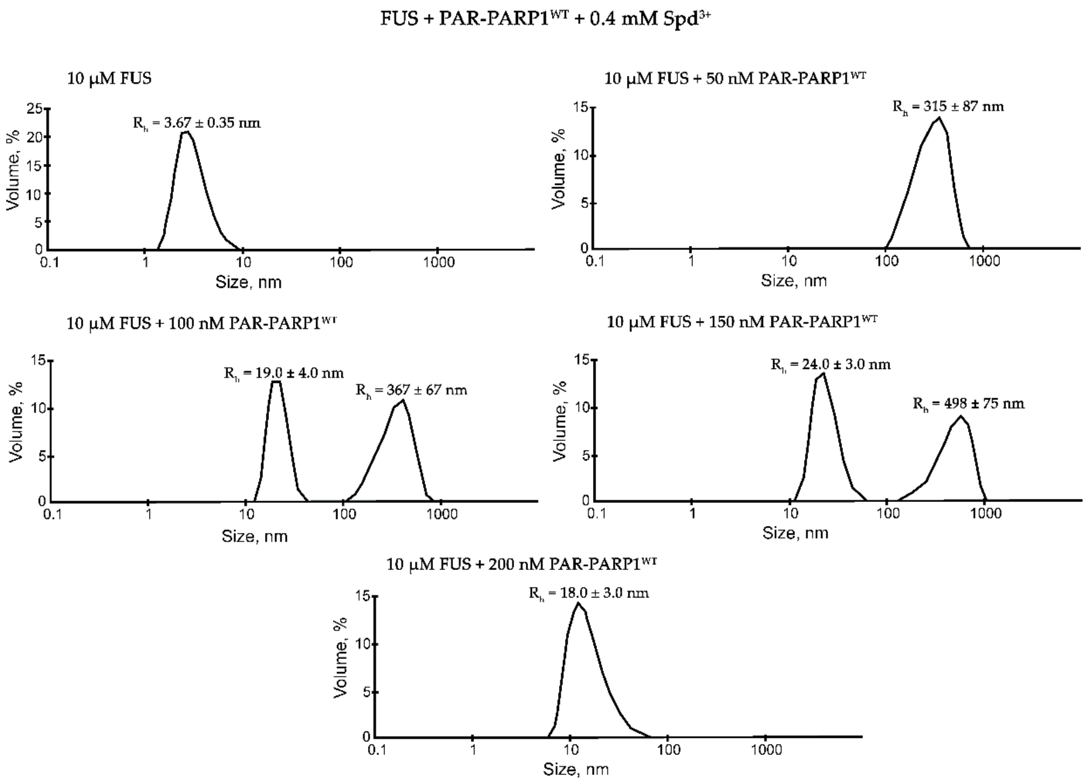

Figure S1d. Typical volume-weighted size distributions for the mixture of FUS with PAR-PARP1^{WT} in the presence of Spd^{3+}. The profiles were obtained by means of experimental autocorrelation functions in the Zetasizer Nano ZS software. The average hydrodynamic radii (R_h) computed from the distributions are presented as well. R_h is the average R_h value estimated from at least three DLS experiments. FUS higher-order structure assays in the presence of PAR-PARP1^{WT} were performed in reaction mixtures consisting of 10 μM FUS, 50-220 nM PARylated PARP1 and 0.4 mM Spd^{3+} in DLS buffer consisting of 25 mM HEPES-NaOH pH 7.5, 200 mM NaCl, 300 mM urea, and 1 mM DTT. The R_h values were measured directly after 1-min incubation of FUS with PAR-PARP1^{WT}.

FUS + PAR-PARP1^{WT} + 0.1 mM Spn^{4+}

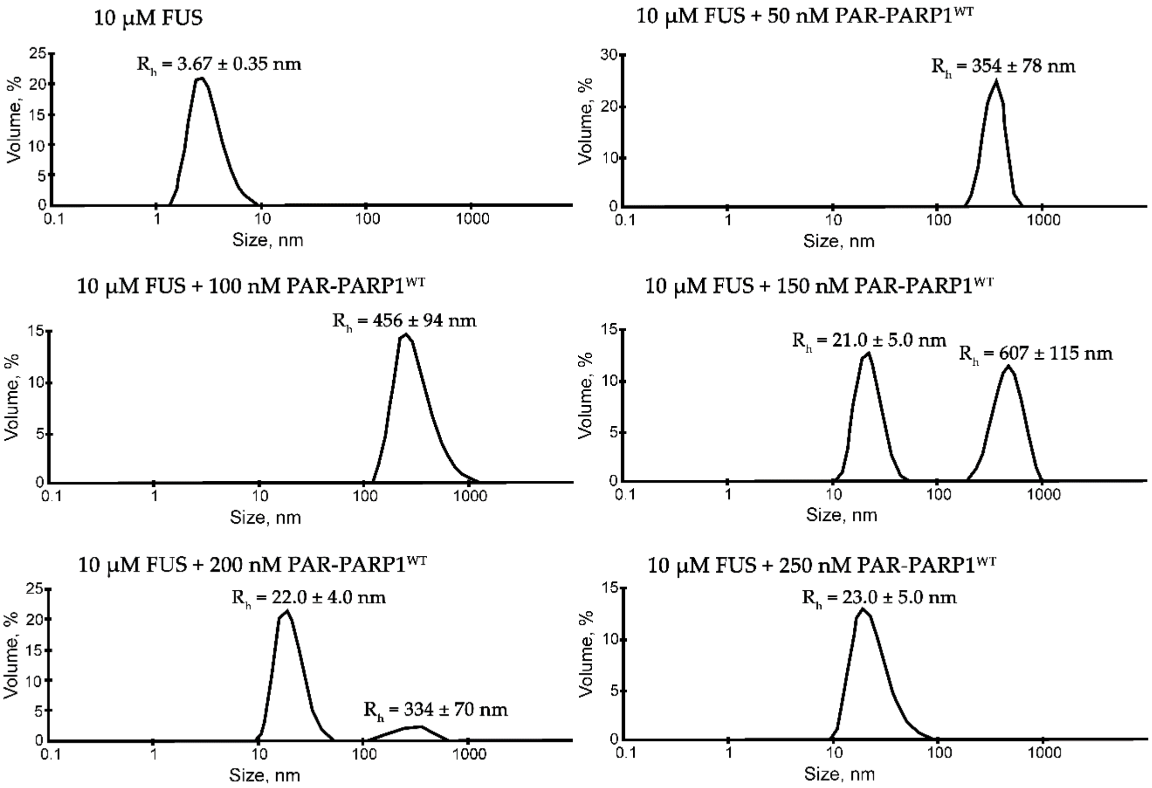

Figure S1e. Typical volume-weighted size distributions for the mixture of FUS with PAR-PARP1^{WT} in the presence of Spn^{4+}. The profiles were obtained by means of experimental autocorrelation functions in the Zetasizer Nano ZS software. The average hydrodynamic radii (R_h) computed from the distributions are presented as well. R_h is the average R_h value estimated from at least three DLS experiments.

FUS higher-order structure assays in the presence of PAR-PARP1WT were performed in reaction mixtures consisting of 10 μM FUS, 50-220 nM PARylated PARP1 and 0.1 mM Spn^{4+} in DLS buffer consisting of 25 mM HEPES-NaOH pH 7.5, 200 mM NaCl, 300 mM urea, and 1 mM DTT. The R_h values were measured directly after 1-min incubation of FUS with PAR-PARP1^{WT}.

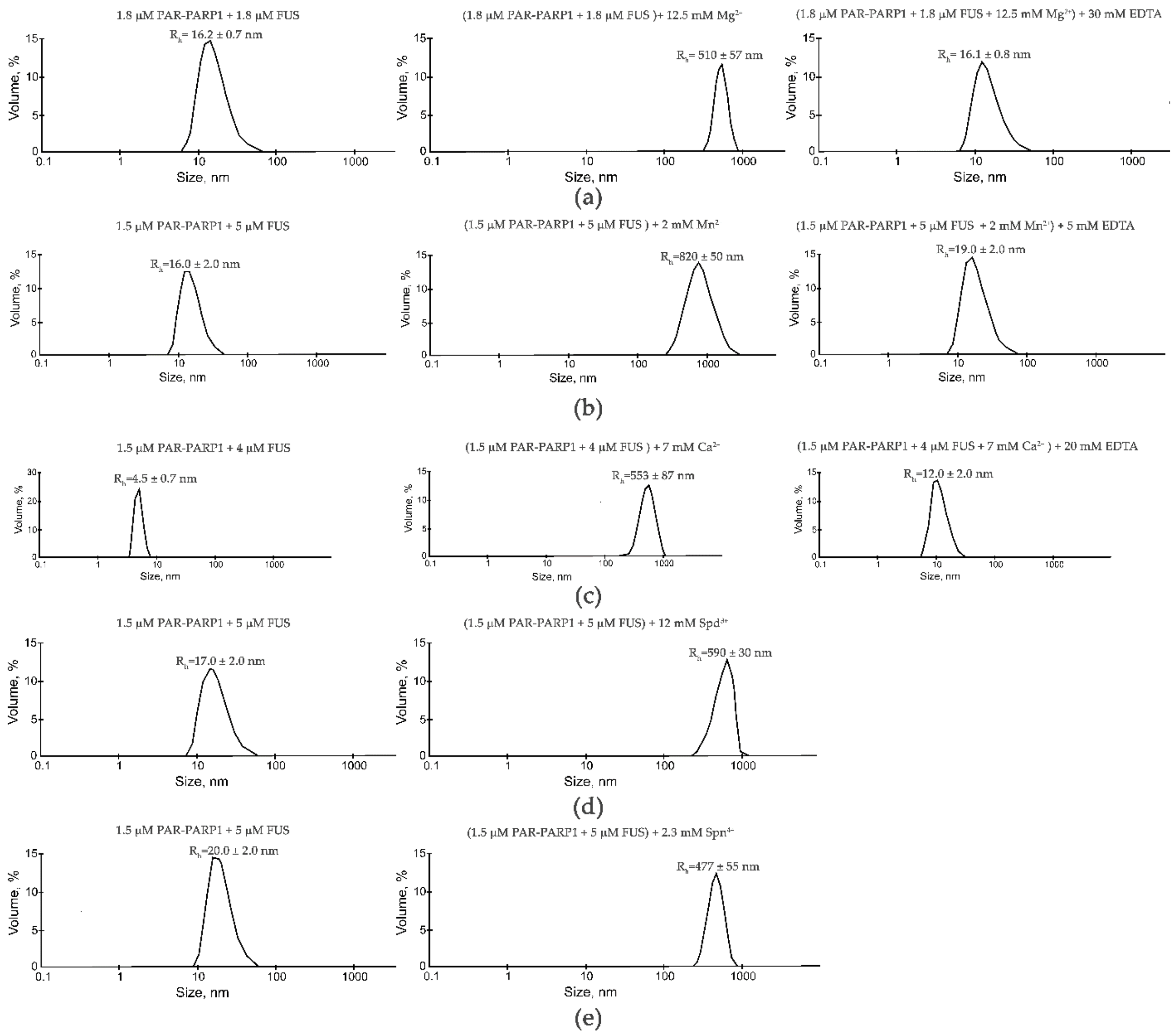

Figure S2. Typical volume-weighted size distributions for the mixture of FUS with PAR-PARP1^{WT} in the presence of millimolar concentration of cations. The profiles were obtained by means of experimental autocorrelation functions in the Zetasizer Nano ZS software. The average hydrodynamic radii (R_h) computed from the distributions are presented as well. Rh is the average Rh value estimated from at least three DLS experiments. FUS higher-order structure assays in the presence of PAR-PARP1WT were performed in reaction mixtures consisting of 4-5 μM FUS, 1.5-1.8 μM PARylated PARP1 and 12.5 mM Mg^{2+} (a), 2 mM Mn^{2+} (b), 7 mM Ca^{2+} (c), 12 mM Spd^{3+} (d) or 2.3 mM Spn^{4+} (e) in DLS buffer consisting of 25 mM HEPES-NaOH pH 7.5, 200 mM NaCl, 300 mM urea, and 1 mM DTT. The Rh values were measured directly after 1-min incubation of FUS with PAR-PARP1^{WT} in the presence of cations. To disrupt FUS - PAR-PARP1 assemblies stabilized by a cation, EDTA to a final concentration of 5 mM (b), 20 mM (c) or 30 mM (a) was added as indicated in figure legends, and R_h was measured in the EDTA-treated samples.

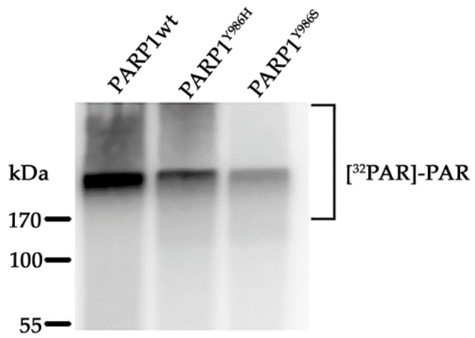

Figure S3. PARylation of PARP1^{WT}, PARP1^{Y986H} and PARP1^{Y986S} according to SDS-PAGE and phosphorimaging. The reaction mixtures (20 μL) consisted of 1 μM PARP1^{WT}, PARP1^{Y986H} or PARP1^{Y986S}, 2 μM DNA duplex (30-mer with a one-nucleotide gap), 200 mM NaCl, 25 mM HEPES-NaOH pH 7.5, 1 mM DTT, 1 mM NAD$^+$, [^{32}P]NAD (1 μCi/100 μL). The reactions were initiated by the addition of NAD$^+$ and were allowed to proceed at 30°C for 40 min. The reactions were stopped by the addition of SDS sample loading buffer and heating for 1.5 min at 97°C and were analyzed by denaturing 10% SDS-PAGE as described elsewhere (Laemmli, 1970). Bands of proteins labeled with [^{32}P]ADP-ribose were visualized and quantified by phosphorimaging on Typhoon FLA 7000 (GE Healthcare, USA) and in the Quantity One Basic software.

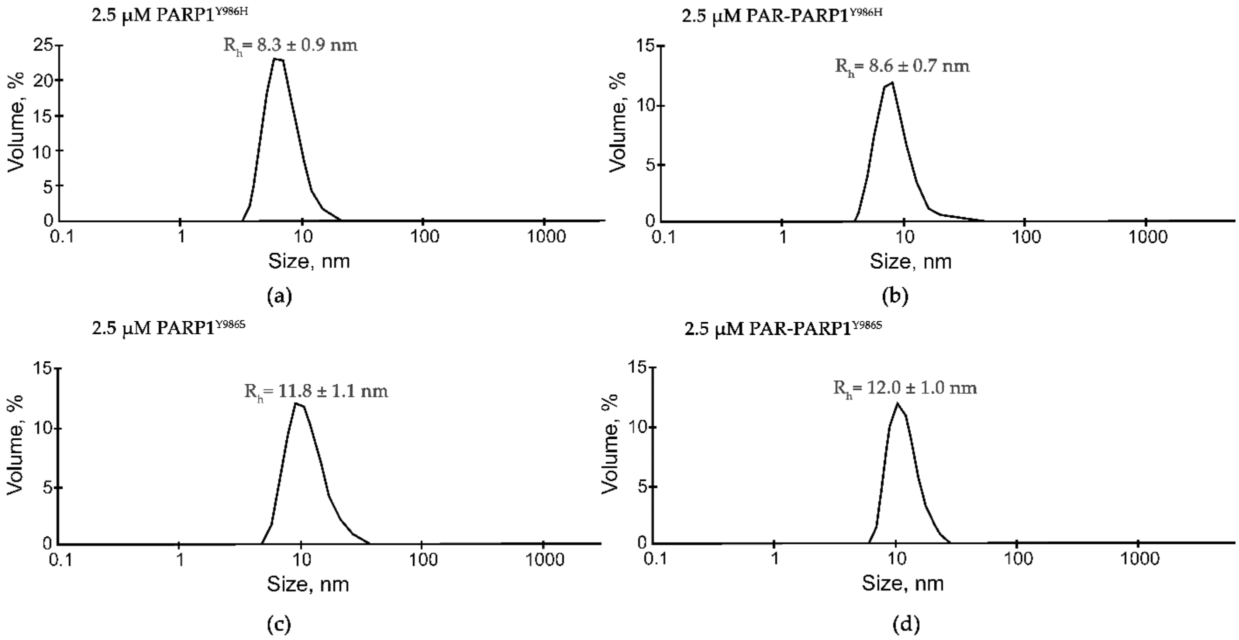

Figure S4. Typical volume-weighted size distributions for PARP1^{Y986H} (**a**), PAR-PARP1^{Y986H} (**b**), PARP1^{Y986S} (**c**) and PAR-PARP1^{Y986S} (**d**). The profiles were obtained by means of experimental autocorrelation functions in the Zetasizer Nano ZS software. The average hydrodynamic radii (R_h) computed from the distributions are presented as well. R_h is the average R_h value estimated from at least three DLS experiments.

PARP1^{Y986H} (**a**) or PARP1^{Y986S} (**b**) hydrodynamic size assays were performed in reaction mixtures consisting of DLS buffer (25 mM HEPES-NaOH pH 7.5, 200 mM NaCl, 300 mM urea, and 1 DTT), 2.5 μM PARP1^{Y986H} or 2.5 μM PARP1^{Y986S} as indicated at the figure legends. The samples were equilibrated for 1 min, and then Rh measurement was performed.

For analysis of PAR-PARP1^{Y986H} (**b**) or PAR-PARP1^{Y986S} (**d**) hydrodynamic size, 2.5 μM PARP1^{Y986H} or PARP1^{Y986S} was incubated with 2.5 μM DNA-gap in a DLS buffer. Samples were equilibrated for 1 min, and then the PARP1 activation was initiated by the addition of NAD$^+$ to a final concentration of 1 mM. The reaction mixtures were incubated at 30 °C for 60 min, and then Rh measurement was performed

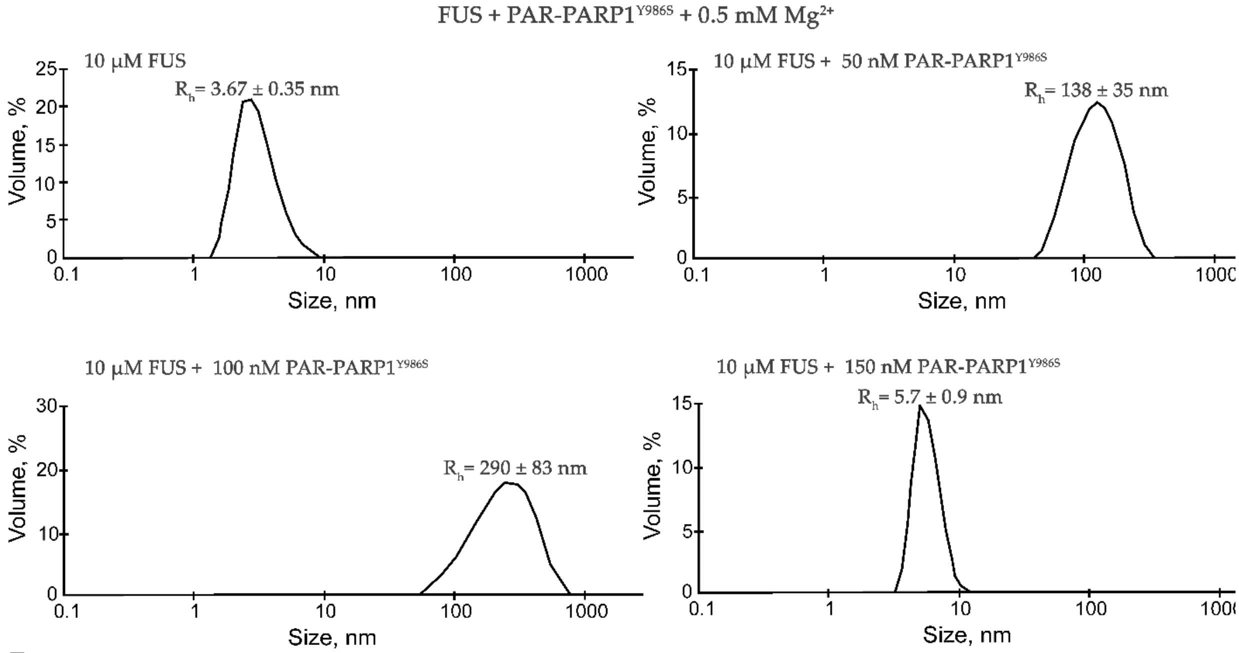

Figure S5a. Typical volume-weighted size distributions for the mixture of FUS with PAR-PARP1^{Y986S}. The profiles were obtained by means of experimental autocorrelation functions in the Zetasizer Nano ZS software. The average hydrodynamic radii (R_h) computed from the distributions are presented as well. R_h is the average R_h value estimated from at least three DLS experiments. FUS higher-order structure assays in the presence of PAR-PARP1^{Y986S} were performed in reaction mixtures consisting of 10 μM FUS, 50-150 nM PARylated PARP1^{Y986S} and 0.5 mM Mg^{2+} in DLS buffer consisting of 25 mM HEPES-NaOH pH 7.5, 200 mM NaCl, 300 mM urea, and 1 mM DTT. The R_h values were measured directly after 3-min incubation of FUS with PAR-PARP1^{Y986S}.

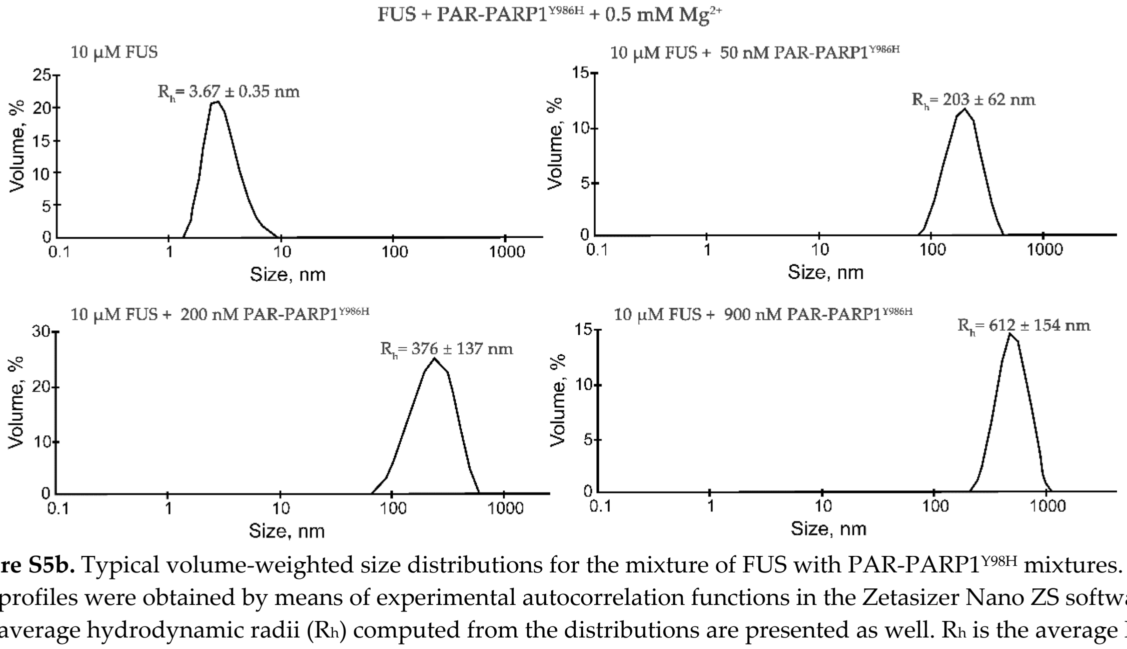

Figure S5b. Typical volume-weighted size distributions for the mixture of FUS with PAR-PARP1^{Y98H} mixtures. The profiles were obtained by means of experimental autocorrelation functions in the Zetasizer Nano ZS software. The average hydrodynamic radii (R_h) computed from the distributions are presented as well. R_h is the average R_h value estimated from at least three DLS experiments. FUS higher-order structure assays in the presence of PAR-PARP1^{Y986H} were performed in reaction mixtures consisting of 10 μM FUS, 50-900 nM PARylated PARP1^{Y986H} and 0.5 mM Mg^{2+} in DLS buffer consisting of 25 mM HEPES-NaOH pH 7.5, 200 mM NaCl, 300 mM urea, and 1 mM DTT. The R_h values were measured directly after 3-min incubation of FUS with PAR-PARP1^{Y986H}.

FUS + PAR-PARP1^{Y986H}

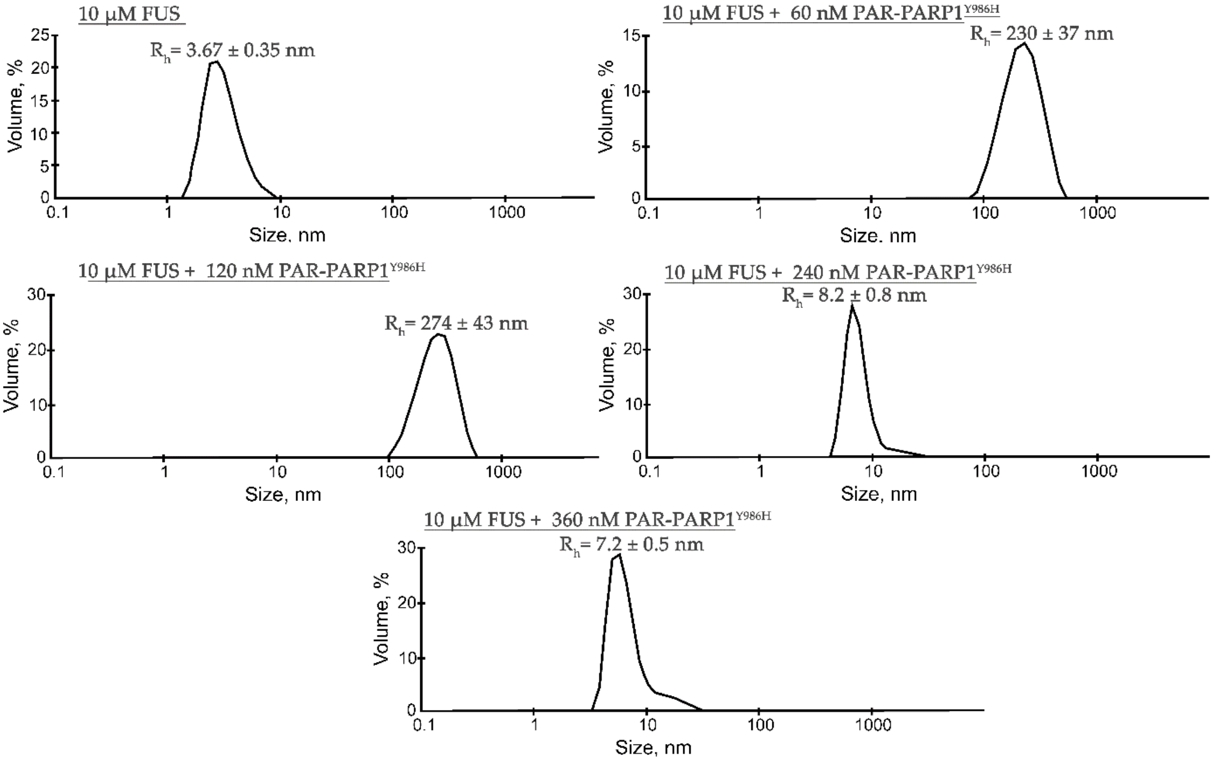

Figure S5c. Typical volume-weighted size distributions for the mixture of FUS with PAR-PARP1^{Y98H}. The profiles were obtained by means of experimental autocorrelation functions in the Zetasizer Nano ZS software. The average hydrodynamic radii (R_h) computed from the distributions are presented as well. R_h is the average R_h value estimated from at least three DLS experiments. FUS higher-order structure assays in the presence of PAR-PARP1^{Y986H} were performed in reaction mixtures consisting of 10 µM FUS, 60-360 nM PARylated PARP1^{Y986H} in DLS buffer consisting of 25 mM HEPES-NaOH pH 7.5, 200 mM NaCl, 300 mM urea, and 1 mM DTT. The R_h values were measured directly after 3-min incubation of FUS with PAR-PARP1^{Y986H}.

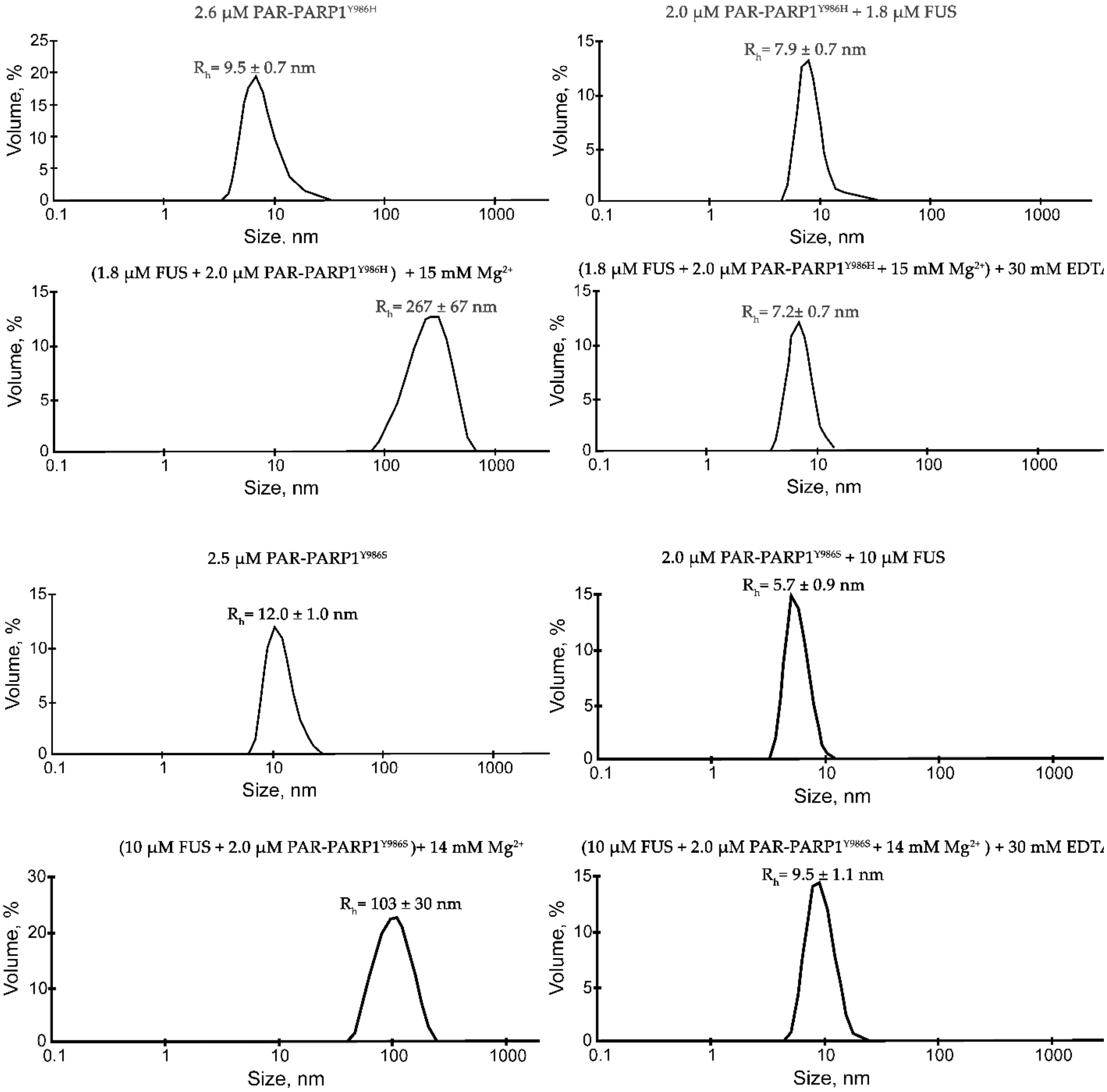

Figure S5d. Typical volume-weighted size distributions for the mixture of FUS with PAR-PARP1^{Y986H}. The profiles were obtained by means of experimental autocorrelation functions in the Zetasizer Nano ZS software. The average hydrodynamic radii (R_h) computed from the distributions are presented as well. R_h is the average R_h value estimated from at least three DLS experiments. FUS higher-order structure assays in the presence of PAR-PARP1^{Y986H} were performed in reaction mixtures consisting of 1.8 μM FUS, 2 μM PAR-PARP1^{Y986H} and 15 mM Mg^{2+} or 10 μM FUS, 2 μM PAR-PARP1^{Y986S} and 14 mM Mg^{2+} in DLS buffer consisting of 25 mM HEPES-NaOH pH 7.5, 200 mM NaCl, 300 mM urea, and 1 mM DTT. The Rh values were measured directly after 1-min incubation of FUS with PAR-PARP1^{Y986H} or PAR-PARP1^{Y986S} in the absence or presence of 15 (14) mM Mg^{2+}. To disrupt FUS - PAR-PARP1 assemblies stabilized by cations, EDTA to a final concentration of 30 mM was added as indicated in figure legends, and R_h was measured in the EDTA-treated samples.

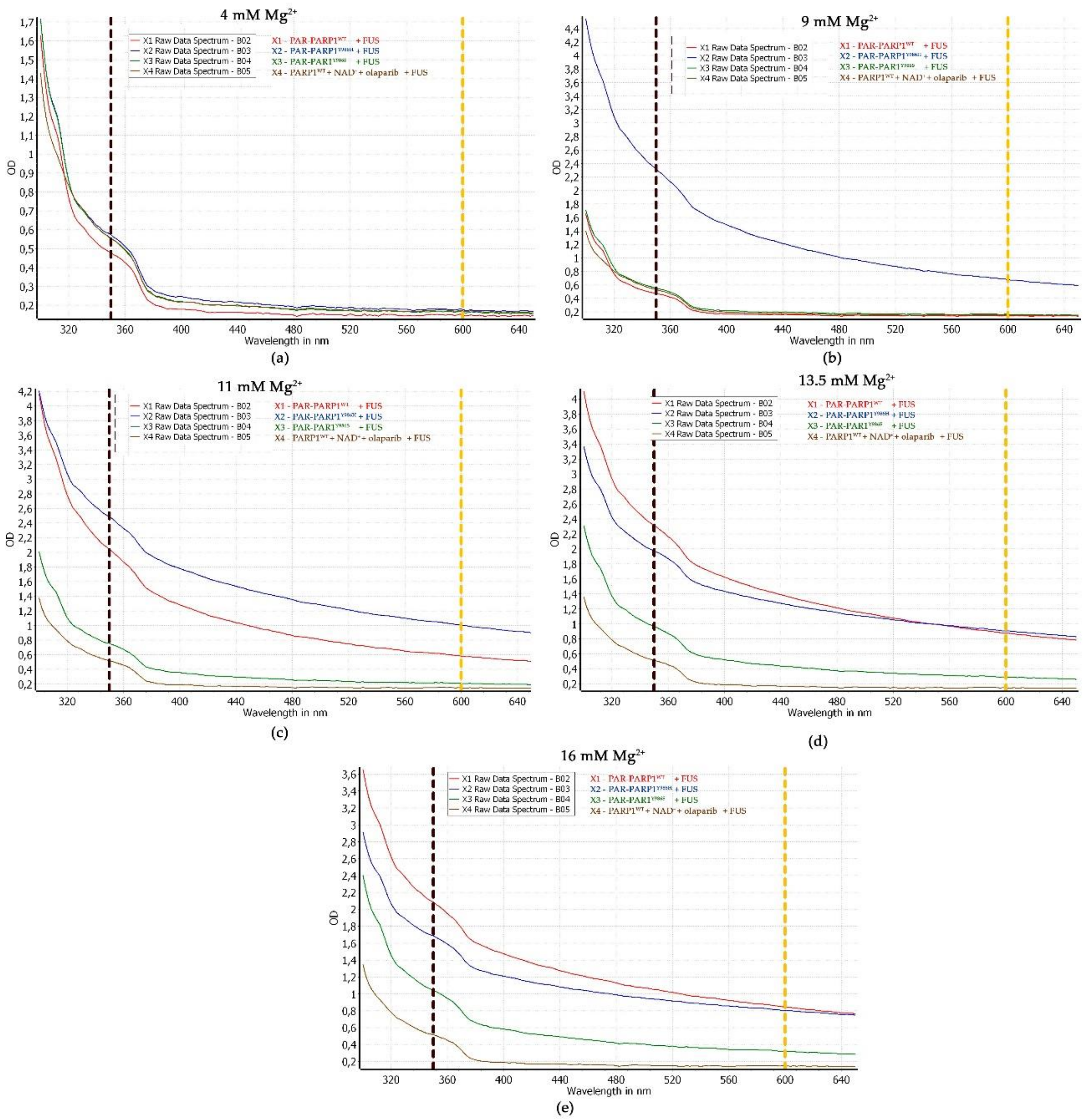

Figure S6. Absorbance spectra of the mixtures consisting of FUS and PARylated PARP1^{WT}, FUS and PARylated PARP1^{Y986H} or FUS and PARylated PARP1^{Y986S} in the presence of different concentration of Mg^{2+}.
2.5 μM PARP1 was incubated with 2 μM DNA-gap and 1 mM NAD$^+$ at 30 °C for 40 min. The reactions were stopped by the addition of olaparib to a final concentration of 200 μM. After that, 1.8 μM FUS was mixed with 2 μM PAR-PARP1^{WT} (red line), PAR-PARP1^{Y986S} (green line) or PAR-PARP1^{Y986H} (blue line) in a buffer consisting of 100 mM NaCl, 20 mM HEPES-NaOH pH 7.5, 300 mM urea and incubated at 30 °C for 5 min. After that, the reactions were supplemented with 4-16 mM Mg^{2+} as indicated in a figure legend, and an absorption spectrum of the solutions was recorded.
The reaction mixture composed of 1.8 μM FUS and 2.5 μM PARP1^{WT} after incubation with 2 μM DNA-gap, 1 mM NAD$^+$ in the presence of 200 μM olaparib and 16 mM Mg^{2+} at 30 °C for 40 min was used as a control (brown line). The absorbance of the protein solution was recorded from 300 to 700 nm in a 96-well non-binding black microplates of transparent bottom (Corning 3881), using a POLARstar Optima multidetection microplate reader (BMG Labtech, Offenburg, Germany). From left to right, two vertical dash lines indicate the position of 350 and 600 nm wavelengths, respectively.

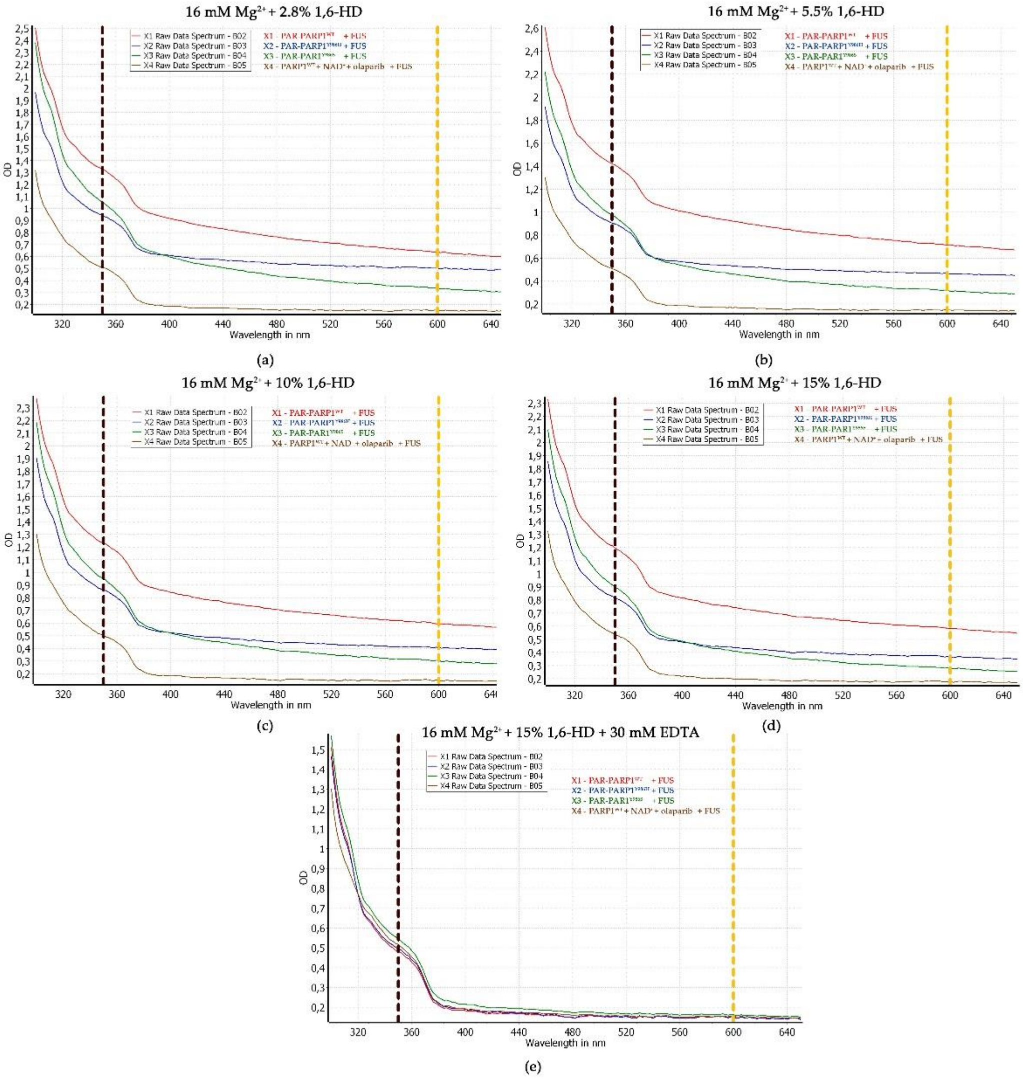

Figure S7. Absorbance spectra of the mixtures consisting of FUS and PARylated PARP1^{WT}, FUS and PARylated PARP1^{Y986H}, FUS and PARylated PARP1^{Y986S} or FUS and PARP1^{WT} in the presence of different concentration of 1,6-hexanediol and (or) EDTA.

(a) Absorbance spectra of the mixtures consisting of FUS and PARylated PARP1 in presence of 16 mM Mg^{2+} after adding of 1,6-hexandiol up to 2.8%.

b) Absorbance spectra of the mixtures consisting of FUS and PARylated PARP1 in presence of 16 mM Mg^{2+} after adding of 1,6-hexandiol up to 5.5%.

c) Absorbance spectra of the mixtures consisting of FUS and PARylated PARP1 in presence of 16 mM Mg^{2+} after adding of 1,6-hexandiol up to 10%.

d) Absorbance spectra of the mixtures consisting of FUS and PARylated PARP1 in presence of 16 mM Mg^{2+} after adding of 1,6-hexandiol up to 15%.

e) Absorbance spectra of the mixtures consisting of FUS and PARylated PARP1 in presence of 16 mM Mg^{2+} after adding of EDTA up to 20-30 mM.

2.5 μM PARP1 was incubated with 2 μM DNA-gap and 1 mM NAD$^+$ at 30 °C for 40 min. The reactions were stopped by the addition of olaparib to a final concentration of 200 μM. After that, 1.8 μM FUS was mixed with 2 μM PAR-PARP1^{WT} (red line), PAR-PARP1^{Y986S} (green line) or PAR-PARP1^{Y986H} (blue line) in a buffer consisting of 100 mM NaCl, 20 mM HEPES-NaOH pH 7.5, 300 mM urea and incubated at 30°C for 5 min. After that, the reactions were supplemented with 16 mM Mg^{2+} (**Figure S6e),** 1,6-hexandiol and EDTA as indicated in a figure legend, and an absorption spectrum of the solutions was recorded.

The reaction mixture composed of 1.8 μM FUS and 2.5 μM PARP1^{WT} after incubation with 2 μM DNA-gap, 1 mM NAD$^+$ in the presence of 200 μM olaparib and 16 mM Mg^{2+} at 30°C for 40 min was used as a control (brown line). The absorbance of the protein solution was recorded from 300 to 700 nm in a 96-well non-binding black microplates of transparent bottom (Corning 3881), using a POLARstar Optima multidetection microplate reader (BMG Labtech, Offenburg, Germany). From left to right, two vertical dash lines indicate the position of 350 and 600 nm wavelengths, respectively**.**
